# Professional development and career-preparedness experiences of STEM Ph.D. students: Gaps and avenues for improvement

**DOI:** 10.1371/journal.pone.0260328

**Published:** 2021-12-16

**Authors:** Shweta Ganapati, Tessy S. Ritchie

**Affiliations:** 1 Mitacs Canadian Science Policy Fellow 2020-21, Natural Sciences & Engineering Research Council of Canada, Ottawa, Canada; 2 Department of Chemistry and Life Science, United States Military Academy, West Point, New York, United States of America; Imam Abdulrahman Bin Faisal University, SAUDI ARABIA

## Abstract

This study presents the experiences of current science, technology, engineering and mathematics (STEM) Ph.D. students and alumni with respect to professional development opportunities in their Ph.D. training. Specifically, it investigates if and how the Ph.D. training supports graduates to pursue non-academic and non-R&D roles, which have become increasingly common career paths post-graduation. A mixed-methods questionnaire was developed to obtain quantitative and qualitative data regarding the graduate school experiences of current Ph.D. students and recent Ph.D. graduates pursuing diverse career paths. The study investigates the values, needs, and conceptions of professional development from the student perspective, as well as the contributions of peers and mentors in graduate school towards their professional development. Experiences of Ph.D. alumni are used to identify the barriers for transitioning to the first job post-graduation and to provide an assessment of the current professional development opportunities in Ph.D. programs. It is reported that although Ph.D. training allowed alumni to develop a robust skillset that includes research, teaching, and scientific writing; some common barriers associated with obtaining a job post-graduation were lack of awareness about career options, limited or no professional networks outside academia, and a lack of preparation and support for non-academic job transitions. Through analyzing the student perspective on various aspects of professional development, the study identifies gaps and avenues for improvement for professional development in Ph.D. training, including increased awareness of diverse career paths for STEM PhDs, increased networking opportunities for PhD students with sectors outside academia, embedding professional development in the PhD curriculum, and others; so that programs can support students in entering the labor market in a variety of careers that extend beyond academia and traditional R&D jobs, using interventions that resonate with the students and meet their needs.

## Introduction

Science, technology, engineering, and mathematics (STEM) graduates represent an important component of the workforce world-wide, and in a knowledge-based economy such as the United States of America (U.S.A.), their contributions to sectors such as healthcare, manufacturing, information services, professional and business services, and others, are key socio-economic drivers [1]. Accordingly, the size of the United States (U.S.) STEM workforce has been growing at a faster rate than that of the non-STEM workforce [1–3]. Within the STEM workforce, doctoral professionals have the lowest long-term (up to 35 years after graduation) unemployment rates compared to master’s and bachelor’s degree holders [4] and they have a higher tendency to work in positions either closely related or at least somewhat related to their STEM background [2].

Despite their socio-economic contributions, in the short term, the employment trends are not entirely favorable for STEM Ph.D. graduates. In 2017, of the 32,359 individuals graduating with a Ph.D. in STEM, in the U.S.; 41% did not have post-graduation commitments in place at the time of graduation [5]. Additionally, postdoctoral researcher positions, which are a common first position post-graduation, are serving as low-paying, high pressure, stop-gap positions for a significant proportion of Ph.D. graduates. These temporary positions do not offer job security and are typically oriented towards training researchers for academic jobs and/ or R&D jobs exclusively [6–12].

It is well-known that the employment landscape of STEM doctorates has undergone some noteworthy shifts in the last few decades. The employment of doctoral STEM professionals in the academic sector especially, in full-time and tenure track-positions has been on a steady decline [4]. Careers outside academia and in non-R&D roles such as those in management, professional and other services have been described as “alternative” paths for STEM doctorates, however Ph.D. graduates pursuing diverse career paths post-graduation are becoming increasingly common [13–19]. In 2017, the employment of STEM doctorates by sector was comparable for academia at 43% (tenure and non-tenure track) and the private sector at 42% (including for and not for profit). In terms of R&D work, less than half of the STEM doctorates (42%) were working primarily in R&D roles in 2017 [20, 21].

While the employment landscape for STEM Ph.D.s has shifted towards non-academic as well as non-R&D jobs, doctoral training has not changed or adapted to this shift in the labor market and continues to train students through an apprenticeship model [22–27]. This mismatch has negative consequences not only for the career advancement of Ph.D. graduates, but also for society itself which is delayed in accessing the socio-economic benefits of the Ph.D.s generated [28, 29].

Currently, STEM Ph.D. programs in the USA primarily emphasize research, peer-reviewed publications, and teaching [30]. Other avenues for professional development (PD) of students may come from mentorship by Ph.D. advisors and other academic faculty members, preparation for conference presentations, and peer networks within graduate school [16, 31–35]. These activities and program elements are well-suited for obtaining jobs in the academic and/ or R&D sectors, but not necessarily for pursuing non-academic and non-R&D roles. Awareness of diverse career paths and achieving PD needed to obtain those jobs immediately post-graduation has not been a focus in most STEM Ph.D. programs [36, 37]. In order to prepare Ph.D. graduates for pursuing diverse career paths, it is important to consider if the current skills, experiences, and overall PD achieved through STEM Ph.D. programs are commensurate with the requirements for these diverse job and career outcomes. According to the National Science Foundation’s (NSF) 2019 Survey of Doctoral Recipients, STEM doctoral training confers transferable skills and competencies to graduates such as analytical thinking, innovation, initiative, and others; however, there is still significant scope for improvement in PD targeted towards careers outside academia [38].

Definitions of PD for doctoral training in the literature have encompassed concepts such as distinguishing “Ph.D. completion skills” which are acquired in the course of meeting program requirements such as research, teaching, and publishing and earning the Ph.D. degree; from “professional skills” which may not necessarily be acquired by meeting program requirements alone and include teamwork, communication to lay people, managing people and budgets, as well as competencies in personal effectiveness, career management and self-promotion. The idea of various tiers of learning for achieving PD, including from the Ph.D. advisor, peers, the academic department within the university, career centers at universities, discipline specific professional associations, and a broader “global village” comprising international and inter-sectoral exposure has also been explored. However, these concepts are derived from reviews of published reports on Ph.D. labor market outcomes and existing practices in training; and do not capture the experiences of Ph.D. students themselves [39, 40].

According to the literature on the current student perspective, PD in Ph.D. training is multifaceted and complex. It encompasses personal and professional values, peer- support and mentorship, experiences emphasizing skill-development, and engagement in activities that promote job readiness [16, 31–35, 41, 42]. Studies capturing the current student experiences of PD in STEM Ph.D. programs are few, but this is a growing field of study in the literature, especially within the biological and biomedical sciences. In order to survey the broader STEM landscape, there is a need to study the experiences of Ph.D. students in a variety of STEM disciplines, including from disciplines which are relatively underrepresented in the literature, such as physical and social sciences, to understand their experiences with PD. The alumni perspective of Ph.D. graduates can also be useful in informing the PD needs of Ph.D. programs. Accordingly, studies have collected feedback from alumni of Ph.D. programs and their experiences in the labor market, as well as the impacts of the Ph.D. training in their career progression. In this regard, the vast majority of studies in the current literature specifically focus on postdoctoral PD, interviewing postdoctoral researchers about their current PD [8, 43]. Studies interviewing graduates who are in diverse job roles outside academia and/ or in non-R&D roles are an emerging area of investigation and there is a need for more studies focusing on this demographic [44, 45]. Moreover, existing studies only document the experiences of Ph.D. graduates post-graduation *after* they have successfully embarked on non-academic and non R&D careers. They do not capture the experiences of students during the transition into such roles or investigate the barriers faced in trying to obtain non-academic/ non-R&D jobs. Understanding this transition and the associated PD and support needed to facilitate it, is crucial to ensure that graduates do not end-up in stopgap positions post-graduation and are able to secure jobs which lead to fulfilling careers and making socio-economic contributions.

This study aims to advance the knowledge on the experiences of current STEM Ph.D. students and alumni with respect to PD in Ph.D. programs and identify the barriers for transitioning to the first job post-graduation. By considering the values, experiences, and needs of both demographics (current Ph.D. students and alumni in diverse career paths), policymakers, university administration and program directors can help students enter the labor market in a variety of careers that extend beyond academia and traditional R&D jobs using interventions that resonate with the students and meet their needs.

## Methods

### Development of the Career Preparation and Professional Development within Doctoral Programs (CPPDDP) questionnaire

A mixed-methods questionnaire was developed to obtain quantitative and qualitative data regarding the graduate school experience of current Ph.D. students and recent Ph.D. alumni. Ph.D. alumni respondents were limited to those who were within 10 years of having graduated with a Ph.D. at the time of answering the questionnaire to improve the accuracy of recall for questions related to their experiences in graduate school. Research questions were generated after literature review and aimed to address five key questions: (i) how do STEM Ph.D. students and recent alumni define professional development (PD), (ii) what forms of PD are/ have been experienced by them during doctoral training, (iii) what do STEM Ph.D. students and alumni want from their careers, (iv) what role do mentors and peers play in PD, and (v) how do Ph.D. alumni envision the future of PD in STEM Ph.D. training. The survey was piloted by three STEM graduate alumni. They were sent the survey link and were asked for feedback regarding syntax, clarity, content, and tested the survey logic for consistency and accuracy. Any issues found during the pilot were resolved before the survey was distributed. The full questionnaire can be found in the S1 File. The questionnaire was submitted to the Institutional Review Board at the U.S. Naval Academy (USNA) and was granted the Institutional Review Board (IRB) approval (USNA.2019.0019-IR-EP7-A).

### Distribution of the questionnaire

The CPPDDP questionnaire was administered through Google Forms. The CPPDDP questionnaire link, which included a description of the research study, was sent to relevant email lists at graduate schools in the United States and distributed to relevant PhD student and alumni groups on social media such as LinkedIn and Twitter. Additionally, graduate school offices and individual departments were contacted through email. The email provided information on the questionnaire and encouraged the distribution among STEM Ph.D. students. The CPPDDP questionnaire was left open from April 2019 through June 2019. A total of 192 responses were recorded, and188 of these were viable for analysis. Answers to the questionnaires were collected anonymously and voluntarily; no incentives were provided for completion of the questionnaire.

### Qualitative data analysis

Short answer responses were analyzed by thematic analysis using the grounded theory framework, specifically inductive coding [46]. An inductive approach, driven by the participants’ responses, was employed to develop a deeper understanding of the STEM Ph.D. professional development experience and its multifaceted nature. First, the authors independently coded the short answer responses. Next, they discussed and refined any codes that were unclear. Finally, the authors re-coded datasets until agreement was met. In the case when a particular response corresponded to multiple codes, it was tagged with each of these codes within the same question. After all responses were tagged according to various codes, the codes were analyzed via axial coding to identify similarities and to draw out concepts which were distilled into an emerging theory.

## Results & discussion

### Section1: Respondent sample and demographics

**Fig 1:**
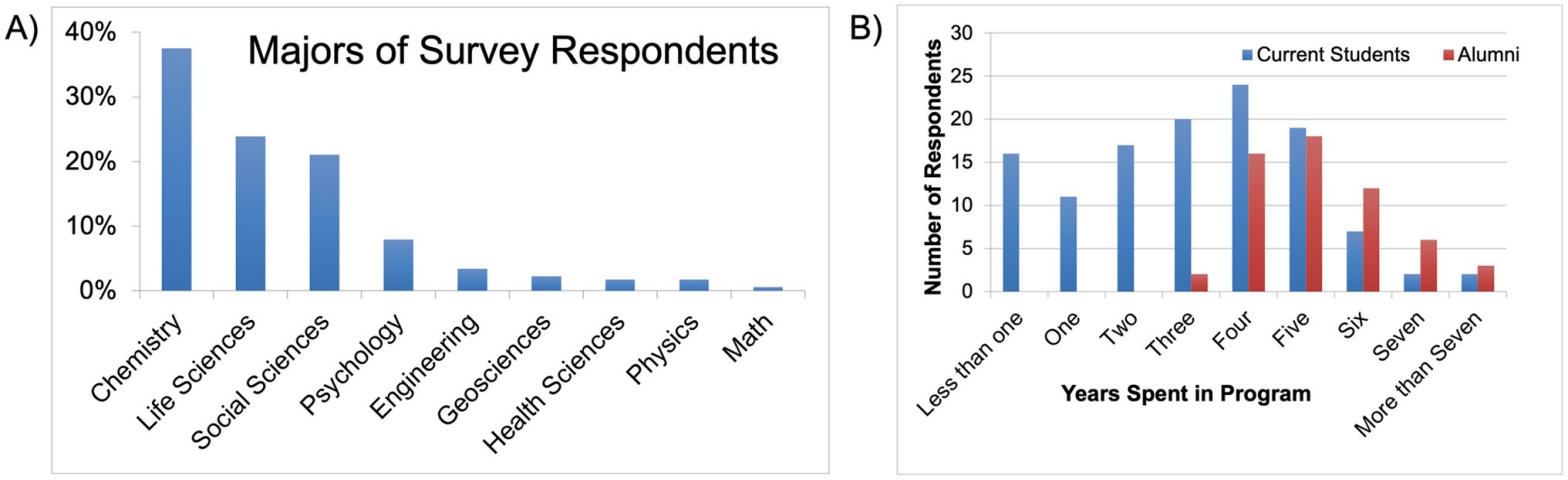
a) Distribution of responses across various STEM majors including psychology and social sciences primarily sociology, education, linguistics, political science, public policy, economics, and others. and b) distribution of responses by number of years spent in Ph.D. program.

The total number of viable responses (n = 188) was refined using the NSF definition of STEM to provide the study sample (n = 176) [4]. This definition includes natural sciences (such as physics, chemistry, and biology), computer and information sciences, social and behavioral sciences (such as psychology, economics, sociology, and political science), engineering, and mathematics. This dataset mostly consisted of responses from current Ph.D. students and recent Ph.D. alumni from the U.S. (n = 164, 93.2%), as well as a small percentage of respondents from non-U.S. institutions (n = 12, 6.8%). Within the sample, 62.5% (n = 110) self-identified as women and 37.5% (n = 66) as men. In terms of disciplinary background, the majority of the sample self-identified as being currently or formerly enrolled in a Ph.D. in chemistry (37.5%, n = 66), followed closely by life sciences (23.9%, n = 42) and social sciences (21.0%, n = 37). The remaining responses were from psychology (8.0%, n = 14), engineering (3.4%, n = 6), geosciences (2.3%, n = 4), health sciences (1.7%, n = 3), physics (1.7%, n = 3) and mathematics (0.6%, n = 1, Fig 1a). Of the set of students who self-identified, 67.6% (n = 119) identified as current Ph.D. students, and 32.4% (n = 57) identified as recent Ph.D. alumni; at the time of answering the questionnaire (Fig 1b). Additional demographic information is available in the S1 File.

### Section 2: Perception and experiences of PD in doctoral training

The second section of this study explores the definition of PD from the perspective of Ph.D. students and alumni; the activities which they consider valuable towards their PD, comparing them to activities they typically experience as part of doctoral training; and their career values. The analysis aims to deepen the understanding of the PD needs of Ph.D. students and alumni, and to identify how they are being met and where there are gaps within the current training.

#### Identifying the components of PD in a doctoral context

An individual’s understanding of PD may develop based on a number of factors, such as past experiences (for example, through prior work experience), learned knowledge (from teachers, parents, peers, and literary sources), and on the basis of certain assumptions, expectations, and personal values. In order to understand the definitions upon which participants would be basing their responses to the questionnaire, they were first asked to share their definitions of PD. The coding of responses and some examples are presented in Table 1.

**Table 1:** Definition of professional development (n = 176).

Code	Description of Code	Example Responses
Skill development (51.7%)	Respondents described both technical/ scientific skills and non-technical skills such as communication, job application/ interview related, and interpersonal skills.	“The opportunities that one has as a graduate student to gain **necessary skills to succeed in the job market, but I don’t refer to just technical skills, I mean networking, CV tailoring**, etc.”
		“It is an **amalgam of scientific and personal skills** essential for career growth.”
		“Activities that develop **skills beyond the normal scope of doing one’s job** (in this case academic research).”
		“Professional development is an opportunity to refine **skills that you will use in your future career**.”
Long-term career growth/ planning (39.2%)	Respondents described career advancement for success post Ph.D. and the ability to grow in one’s profession over time.	“Learning **how to advance in one’s chosen field**. This would include, but is not limited to interview strategies, networking tips, resume editing, etc.”
		“Becoming more prepared to **face future shifts and changes** in your profession”
		“Making yourself **ready for your interested career path—each career is different;** so, the preparation of each individual has to have different approaches.”
Gaining knowledge (26.1%)	Respondents described gaining knowledge about diverse career paths, the job market, norms/ culture of their field, and knowledge that would be directly applicable in their professional work.	“Professional development for students has two phases, in my opinion. First, students need to **gather information about career paths and decide what they want to pursue**. That’s where a lot of the GSO’s (graduate student organization) events would be helpful, I think. Once you have a clear path in mind, however, professional development gets more specific and more diverse. For me, it was learning how to write a research and teaching statement, **learning about the academic job market**, etc.”
		“Programming specifically **designed to inform participants about different career paths** and the process towards being successful in the specified career paths”
Getting a job (19.3%)	Respondents specified a goal of PD as getting a job upon graduation.	“Ability to successfully apply, interview, and **receive an employment offer**.”
		“To successfully **transit from school to workplace**”
Gaining experience (11.4%)	Respondents described gaining experiences which they could add to their C.V.s, would help in getting a job, and would help in advancing in their careers.	“Intentional training or **experience that prepares a person for greater effectiveness or competency in their profession**.”
		“Investing time to improve and learn new skills directly related to your future career aspirations. The most useful professional development skills are **experiences that may be added to your C.V. when applying to a job**..”
Becoming a competitive job applicant (9.7%)	Respondents described wanting to stand out in the job market.	“**Becoming a more competitive job applicant**.”
		“Preparing to **enter the job market**.”
Expanding network (6.8%)	Respondents described expanding their professional networks both within and beyond academia and also highlighted the quality of interactions and connections.	“Developing skills in non-science disciplines and **relationships with those in your field but outside of regular contacts**.”
		“finding ways to **increase your quality of interaction and connections with other professionals in your field** in order improve your chances of success and getting a job”
Self-improvement (4.0%)	Respondents described PD as a holistic personal experience, beyond job search and career advancement.	“Work towards **making yourself a better worker, thinker, leader**”
		“Proceeding on **intellectual journey**”
		“train me to be a **good researcher, teacher, scholar, citizen**, etc.”

Consistent with existing definitions of PD in the literature, respondents in this sample also viewed PD as multifaceted, involving gaining skills (51.7%), knowledge (26.1%), experiences (11.4%), professional networks (6.8%), and personal growth (4.0%). PD was also defined as a process with both short-term goals such as becoming a competitive job applicant (9.7%) and obtaining a job (19.3%); and long-term goals such as career development (39.2%, Section 7, Q1 in S1 File).

Acquiring skills was the most highlighted theme in the responses, and respondents described both technical skills (such as research skills, instrumentation techniques, technical writing) and non-technical skills (such as communication, job application/ interview related, and interpersonal skills). Similarly, gaining knowledge was mentioned both, in the context of technical knowledge within specific scientific disciplines; and also, knowledge about diverse career paths, the job market, and the norms/ culture of various fields. The description of experiences resulting in PD included experiences directly corresponding with the apprenticeship model of academic training (such as research, teaching, publishing papers, and presenting at conferences), along with experiences which would stand out in a curriculum vitae (C.V.), improve employability, and would help in advancing their careers in non-academic roles. The responses indicated that STEM Ph.D. students and graduates were looking for PD activities that would lead to tangible results and “C.V.-worthy” experiences which would be viewed favorably when applying for jobs. In this case, “job-readiness” was not limited to participating in PD activities that would promote transferable skills which are valuable; it also included having demonstrated experience in applying those skills. Respondents who mentioned expanding their professional networks as a component of PD, specified both networks within and beyond academia.

The emphasis on PD as a means to facilitate transition from graduate school to obtaining a job was evident in the inclusion of the following in the definition; job application and C.V. skills, knowledge of diverse career paths, having experiences relevant to obtaining a job, building networks within and beyond academia, wanting to stand out in the job market, and meeting the goal of obtaining a job post-graduation. It was also notable that for a large percentage (39.2%) of the respondents, PD extended beyond obtaining a job, and included long term career planning, including in diverse career paths beyond academia.

When taken together, the critical elements for PD as perceived by STEM doctoral students were found to be as follows:

### Professional development allows one to gain skills and knowledge which can facilitate the procurement of tangible results and CV-worthy experiences that will assist in becoming competitive for the job market and ultimately, career advancement in a diverse range of paths

Student perceptions regarding PD are useful to programs for informing interventions that show accountability, facilitate job transitions for students, and improve and maintain program retention.

#### PD Activities in doctoral training

In order to identify gaps between PD experiences which Ph.D. students and alumni considered valuable, versus activities they typically experienced as part of their Ph.D. training; a list of 18 activities was developed by the authors, based on the “tiers of learning” identified by Nerad^17^ (Table 2).The authors identified four categories or tiers based on their proximity to Ph.D. program requirements (Tier 1) on one end, and post-graduation job-attainment (Tier 4) on the other. The categories were designed to include activities that most support the student’s direct context, i.e., the Ph.D. lab, department, or university versus activities which are further removed from this direct context such as professional networks and events, job-searching, and networking. The 18 activities presented as options were selected to represent each of the four tiers, with 4–5 activities identified for each tier, based on examples in the literature, and supplemented with activities that would be broadly applicable to diverse career paths [47]. The tiers were as follows; Tier 1: doctoral program requirements (activities 14–18 in Table 2), Tier 2: engagement with the broader academic community beyond one’s research group (activities 9–13 in Table 2), Tier 3: expanding professional network beyond academia (activities 5–8 in Table 2), and Tier 4: job search-related activities (activities 1–4 in Table 2).

**Table 2:** Table of activities experienced in graduate school, categorized into four tiers.

Activity Number	Professional Development Activity
**Tier 4: Job Search**	
1	Attending an interview skills training workshop
2	Getting your resume reviewed by a professional in your field/ a career coach
3	Doing an internship while in graduate school
4	Attending job fairs and company events hosted on your university campus
**Tier 3: Expanding Professional Network Beyond Academia**	
5	Conducting informational interviews with professionals in your field
6	Attending career seminars where professionals come to discuss their career path
7	Serving as a student leader in your department/ at the University
8	Attending receptions at conferences and meetings to form new connections
**Tier 2: Engagement with Broader Academic Community**	
9	Participating in “3-minute thesis” competitions across disciplines
10	Attending a thesis writing workshop
11	Presenting at a national conference
12	Performing educational outreach efforts at high schools or for young children
13	Presenting at a small conference hosted at your university with mostly intra-university participation
**Tier 1: Doctoral Program Requirements**	
14	Publishing in peer-reviewed journals
15	Mentoring an undergraduate/junior graduate student in your research lab/ department
16	Learning a new scientific skill (e.g.: instrumentation technique, computer language, software)
17	Helping lab-mate advance their research/ contributing to their scientific publication
18	Serving as teaching assistant

Survey respondents were presented with 18 options of potential PD activities (randomized and not presented by tier) and were asked in two series, first regarding which activities they considered to be “valuable” PD activities and then which of the activities they had actually done as Ph.D. students (Section 7, Q2 and Q3 in S1 File, respectively). The top 3 activities that respondents valued were:

- Activity 2 (Tier 4: job search-related): “Getting your resume reviewed by a professional in your field/ a career coach” (84.4%)
- Activity 11 (Tier 2: broader academic community related): “Presenting at a national conference” (79.3%)
- Activity 14 (Tier 1: doctoral program related): “Publishing in peer-reviewed journals” (77.1%)

In comparison, the top activities that students and alumni actually did in graduate school were:

- Activity 16 (Tier 1: doctoral program related): “Learning a new scientific skill such as an instrumentation technique, computer language, or software” (81.0%)
- Activity 18 (Tier 1: doctoral program related): “Serving as teaching assistant” (78.8%)
- Activity 17 (Tier 1: doctoral program related): “Helping a lab-mate advance their research project and/ or contributing to their scientific publication” & Activity 11 (Tier 2: broader academic community related): “Presenting at a national conference” (67.0%)

**Fig 2:**
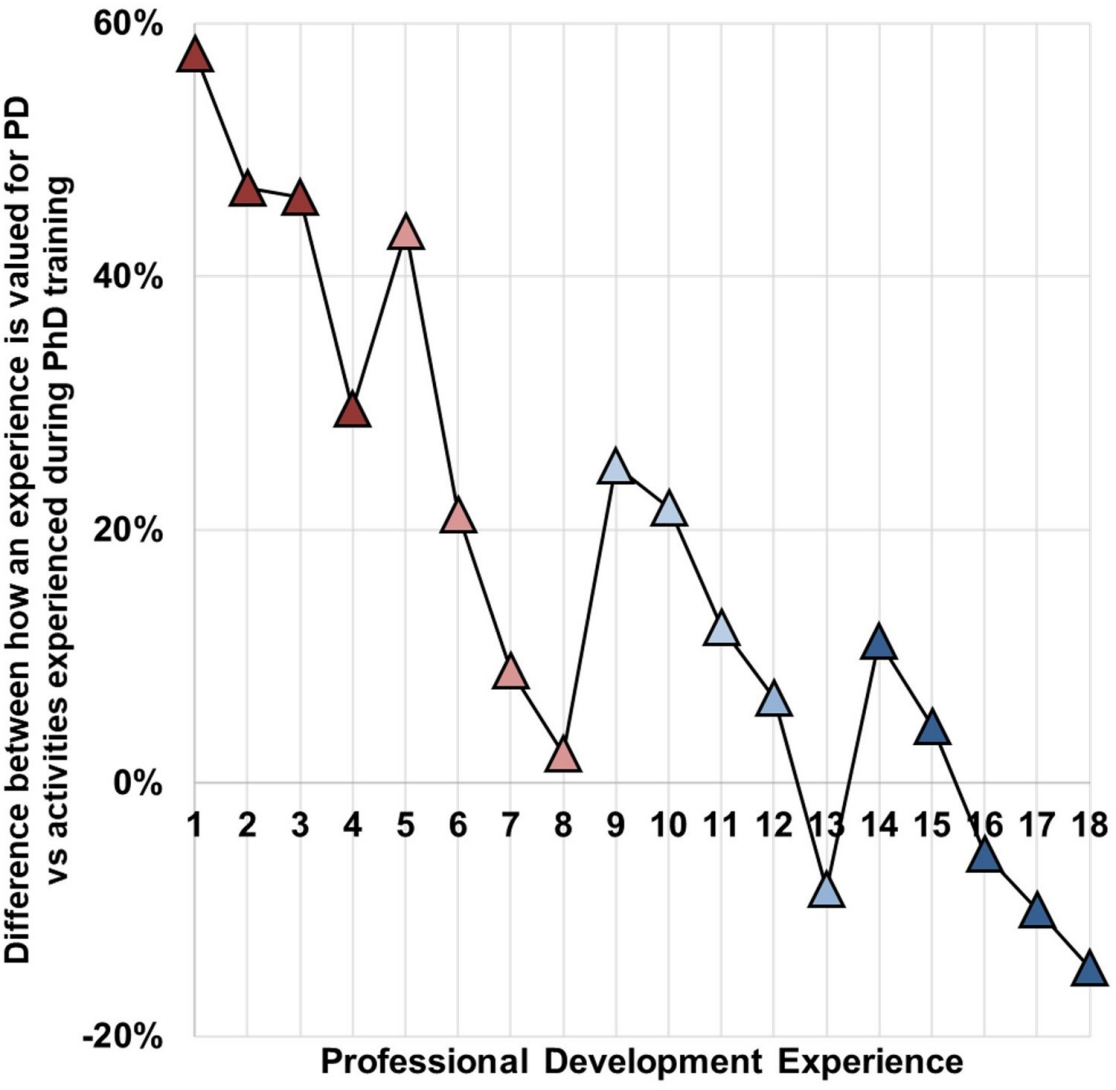
Plot of PD experiences during Ph.D. training according to discrepancy between what Ph.D. students valued for their PD vs. what they actually experienced (n = 176). Activities presented in Table 2 are color coded based on the student’s direct PD context. Activities marked red (greatest discrepancy) are related to job search (Tier 4, dark red, 1–4) and expanding professional network beyond academia (Tier 3, light red, 5–8) and activities marked blue (least discrepancy) are related to doctoral program requirements (Tier 1, dark blue, 14–18) and the broader academic community (Tier 2, light blue, 9–13).

While the top three activities valued by the respondents are distributed among different categories such as job search (Tier 4), academic community (Tier 2), and program related (Tier 1); the activities most commonly experienced by the respondents were mostly concentrated in the program related Tier 1. Activity 11 (“Presenting at a national conference”) was the exception, being a highly valued activity, which was also commonly experienced by Ph.D. students. In order to analyze the trends for which activities had the highest discrepancy between being valued for PD but not being typically experienced in graduate school, the difference in the percentage of respondents who valued each activity for PD versus respondents who actually experienced these activities for each of the 18 options presented (Fig 2) was plotted. As can be observed from Fig 2, the highest discrepancies, between 40–60%; were observed for Tier 4 ‘job related activities’ and Tier 3 ‘expanding network beyond academia related activities. Specifically, these activities were:

- Activity 1 (Tier 4: job search-related): “Attending an interview skills training workshop”
- Activity 2 (Tier 4: job search-related): “Getting your resume reviewed by a professional in your field/ a career coach”
- Activity 3 (Tier 4: job search-related): “Doing an internship while in graduate school”
- Activity 5 (Tier 3: expanding network beyond academia related): “Conducting informational interviews with professionals in your field”

Interestingly, these were all activities which would most likely benefit the student in their job search, with fewer direct benefits to the student’s local environment (i.e., the Ph.D. program).

The next tier of activities which had a discrepancy of 20–40% between being valued and experienced, were also mostly removed from the direct context of the Ph.D. program and were related to either job search (Tier 4), expanding network beyond academia (Tier 3), or to the broader academic community (Tier 2). These activities were as follows:

- Activity 4 (Tier 4: job search-related): “Attending job fairs and company events hosted on your university campus”
- Activity 6 (Tier 3: expanding network beyond academia related): “Attending career seminars where professionals come to discuss their career path”
- Activity 9 (Tier 2: broader academic community related): “Participating in “3-minute thesis” competitions across disciplines”
- Activity 10 (Tier 2: broader academic community related): “Attending a thesis writing workshop”

Following this trend, the lowest discrepancies (between 0–20%) between activities valued and experienced in graduate school were for activities that were most closely related to the academic context (i.e., the research lab, department or university), such as:

- Activity 11 (Tier 2: broader academic community related): “Presenting at a national conference”
- Activity 12 (Tier 2: broader academic community related): “Performing educational outreach efforts at high schools or for young children”
- Activity 14 (Tier 1: doctoral program related): “Publishing in peer-reviewed journals”
- Activity 15 (Tier 1: doctoral program related): “Mentoring an undergraduate/junior graduate student in your research lab/ department”.

Activity 11 (“Presenting at a national conference”) and 14 (“Publishing in peer-reviewed journals”) were the most highly valued activities which were also commonly experienced by students in supporting their PD within the categories of connecting with the broader academic communities and meeting the program requirements.

Interestingly, activities which students commonly experienced in graduate school but did not value for their PD, with negative discrepancies between 0 to -20%, were mostly related to program requirements. Such as:

- Activity 13 (Tier 2: broader academic community related): “Presenting at a small conference hosted at your university with mostly intra-university participation”
- Activity 16 (Tier 2: broader academic community related): “Learning a new scientific skill such as an instrumentation technique, computer language, or software”
- Activity 17 (Tier 1: doctoral program related): “Helping a lab-mate advance their research project and/or contributing to their scientific publication”
- Activity 18 (Tier 1: doctoral program related): “Serving as teaching assistant”

These data point to some important misalignments in the areas of job-search and networking (beyond academia) related PD opportunities within Ph.D. training, as perceived by Ph.D. students and alumni.

#### Career values of Ph.D. students

**Fig 3:**
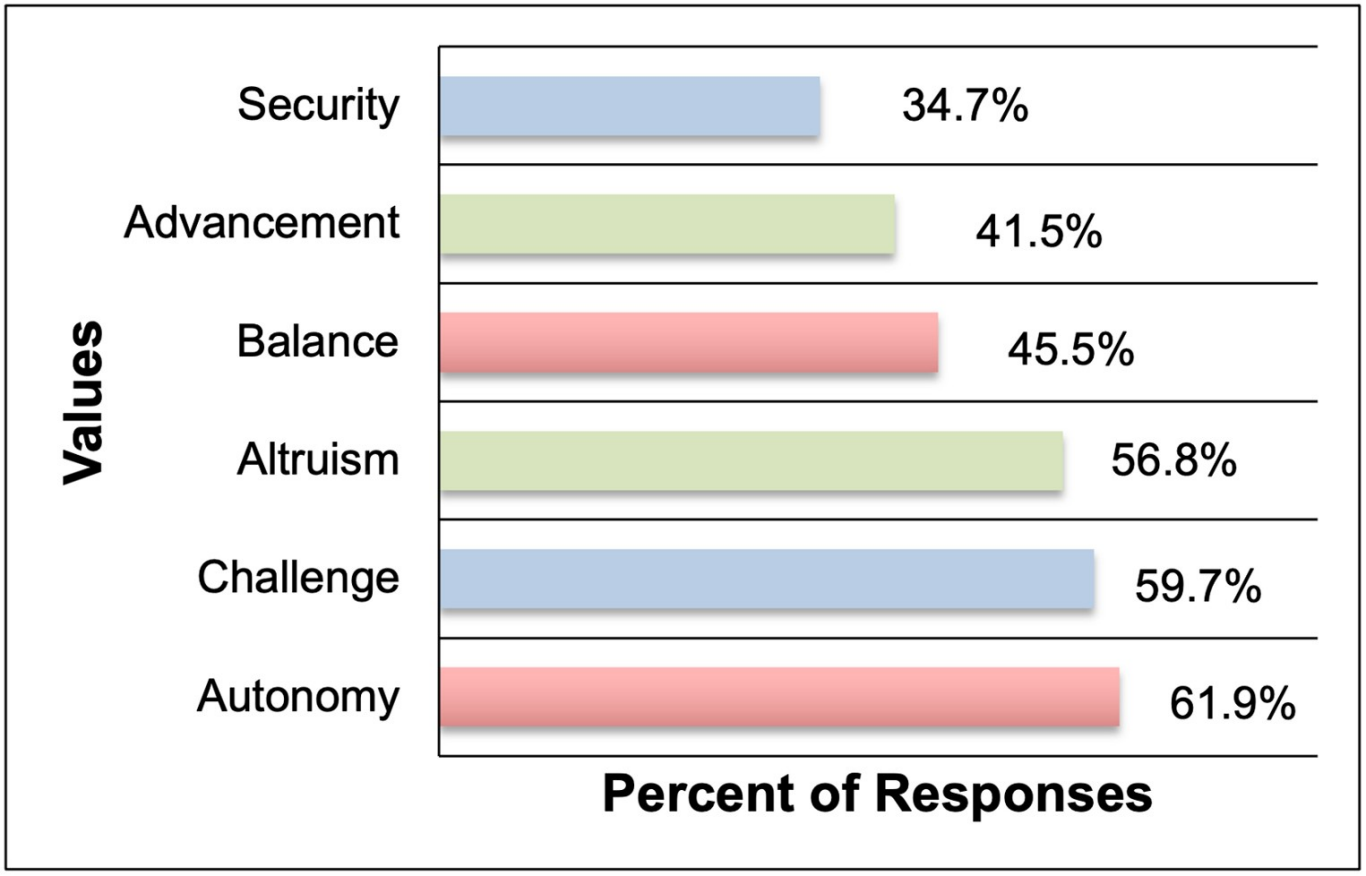
Career values of Ph.D. students and alumni (n = 176), when asked to select top three preferences. The matching colors indicate values that are often conceptualized in a dichotomous manner.

In keeping with the theme of understanding the values of Ph.D. students and recent alumni, survey participants were asked what they valued from their careers. The list of options was generated based on the set of six core values adopted by the American Chemical Society, designed to understand student preferences. These are: (i) advancement, (ii) autonomy, (iii) challenge, (iv) security, (v) balance, and (vi) altruism [48, 49]. These values have been conceptualized in a dichotomous manner: advancement versus altruism, autonomy versus security, and challenge versus balance [50]. The authors provided two examples corresponding to each of these six core values (in a randomized order), a total of twelve options; from which participants were asked to select the top three choices of what they valued the most in their current/ future careers. The list of options provided and percentage responses for each are detailed in the S1 File (Section 7, Q4 in S1 File). When the results were totaled, it was found that STEM Ph.D. students and alumni valued autonomy (61.9%) the most, followed by challenge (59.7%) and altruism (56.8%), as shown in Fig 3. Within these top values, the specific examples chosen were always skewed in favor of one of the 2 examples provided for each value. Nearly 69.7% of the responses for autonomy corresponded to the option “**Independence to direct projects and/or make decisions regarding projects**” as opposed to “Supervising and/or leading a team”, for challenge, 75.2% of the respondents selected “**Performing intellectually demanding tasks**” as opposed to “Performing new and different tasks constantly” and finally 81.0% of those who selected an example within the category of altruism selected “**Doing work that makes a positive difference in society**” as opposed to “Helping less privileged members of society”.

With regard to their careers, Ph.D. students valued roles which would allow for autonomy, challenge, and altruism. Given the nature of research and doing original work, it is not surprising that this demographic chose challenge over balance. It is also worth considering if the choice of autonomy over security plays a role in Ph.D.s remaining in temporary research roles like postdoctoral positions, over taking up other roles where job security may come at the cost of autonomy (such as working for the government or private sector in a research role). It is interesting to note that altruism or the idea of doing work that serves society, is a key driving force for Ph.D. alumni and students and was chosen over advancement. This inclination towards altruism further underscores the value of utilizing this highly skilled and motivated workforce for the betterment of society.

### Section 3: Mentorship & peer support during doctoral training

The third section of this study focuses on the types of professional relationships which Ph.D. students and alumni have access to during their doctoral training. It aims to identify if and how these relationships, specifically with mentors and peers, play a role in the PD of Ph.D. students and alumni.

#### Mentorship and career advice

**Fig 4:**
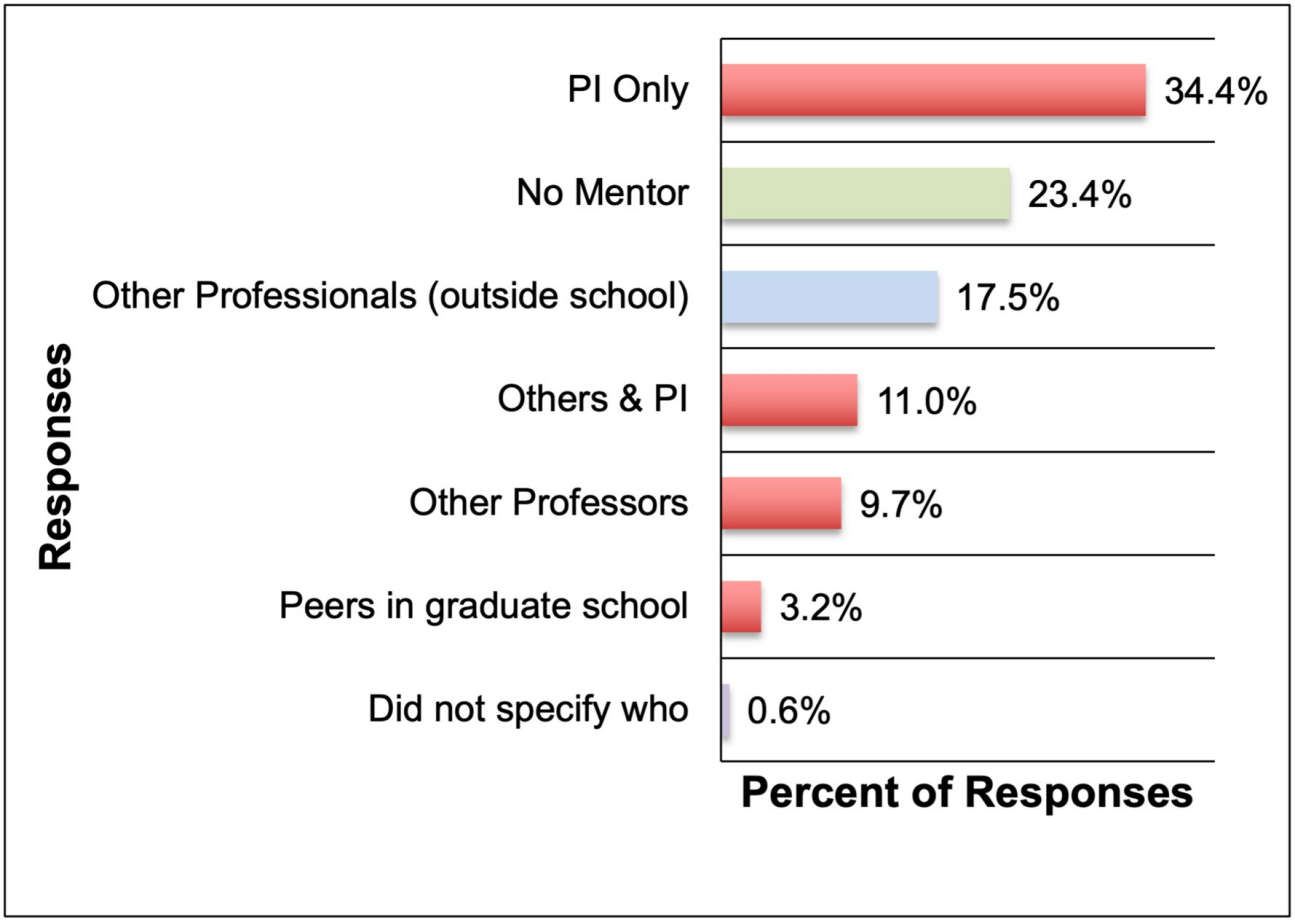
Types of professional mentors of Ph.D. students and alumni (n = 154). The responses in red indicate mentors who are present within the doctoral program.

When asked if the respondents (current Ph.D. students and alumni) currently had a professional mentor and to describe how this mentor was related to them (e.g.: P.I., senior colleague, peer, etc.), a total of 154 responses were received. About a third of the respondents (34.4%) named their P.I. (principal investigator/ advisor of their Ph.D. thesis research projects) as their only professional mentor and an additional 11.0% mentioned their P.I. as at least one of their mentors. Considering that a large percentage of Ph.D. graduates do not end up working in the academic sector in the long term, having greater exposure to professionals in non-academic and non-R&D roles would improve their access to career advice and guidance for a wide range of career paths. It was also disappointing to learn that about a quarter of the respondents (23.4%) did not have any professional mentor. Of the remaining respondents, 9.7% and 3.2% respectively cited that their mentors were either professors (other than their P.I.) or peers in academia. Only 17.5% answered that they had professional mentors who were professionals outside academia (Fig 4). These responses strongly indicate a mentorship gap in Ph.D. training for the purpose of career preparedness, especially for non-academic/ non-R&D careers.

In addition to learning about the availability of professional mentors for Ph.D. students and alumni, the authors were interested in understanding their preferences for mentorship and career advice, if provided options. Specifically, whose advice would Ph.D. students and alumni value and what factors influence this preference. The authors explored whether Ph.D. students and alumni preferred seeking professional advice from entry level versus senior professionals, and additionally, if they had a general preference for reaching out to alumni of their own academic programs. When asked, “Of the following options, whom do you most prefer talking to for career-related advice”, and provided with four options, 174 responses were received. The two most popular responses were senior professionals (43.1%) followed by entry-level professionals (32.8%). Senior professional alumni (13.2%) and entry-level alumni (10.9%) were the least preferred (Section 7, Q5 in S1 File). The preference for senior professionals was attributed to their experience and breadth of knowledge, whereas the preference for entry level professionals was attributed to their relatability and the currentness/ relevance of the information which they would provide (Section 7, Q6 in S1 File). For the respondents who expressed preference for alumni (both senior and entry-level), relatability emerged as the key reason for wanting to connect with alumni. While not a top theme, one that was mentioned several times by those who preferred approaching senior professionals, was that they would most likely be in a position to hire someone. Several respondents specified that if they were looking for advice, they would approach an entry-level professional and if they were looking for a job, they would approach a senior-level professional.

Taken together, the mentorship gap in Ph.D. training for the purpose of career preparedness and guidance needs to be filled by exposure to professionals from various sectors beyond academia with a mix of early career and senior professionals. While some universities have strong alumni networks, this may not be sufficient to meet the needs of Ph.D. students. It is also worth examining if the training provided by P.I.s can be considered as mentorship, especially for the purpose of career preparedness for non-academic and non-R&D career paths.

#### Role of peer-support

**Fig 5:**
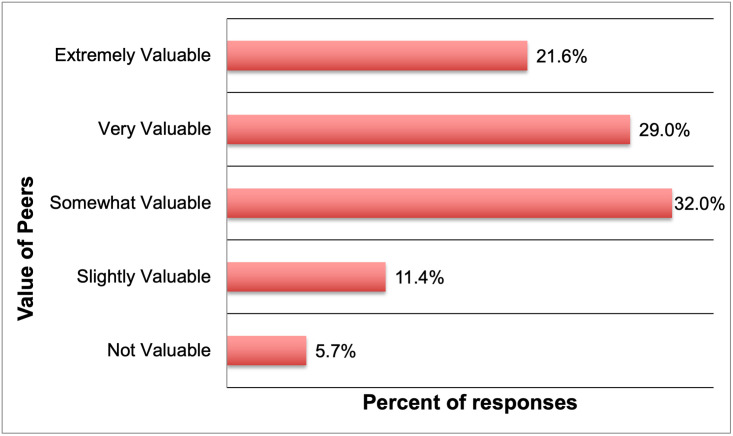
Value of peer networks in learning about PD opportunities (n = 175).

The questions in this section were designed to capture the interactions between peers in graduate school and especially, to glean if and how peers play a role in the PD of Ph.D. students [34, 51]. Upon being asked “How valuable are/ were your peers (from the Ph.D. program) when it involves/ involved learning about new professional development opportunities”; 175 responses were received. As shown in Fig 5, about half (50.6%) answered that their peers were either extremely or very valuable in helping them learn about PD opportunities. Of the remaining, 32.0% answered that their peers were somewhat valuable and 11.4% answered that their peers were at least slightly valuable in this regard. Only 5.7% of the respondents stated that their peers were not valuable in learning about PD opportunities.

When asked to elaborate why respondents selected specific options in response to the previous question, 133 responses were received, which provided insights into how peers help each other in their PD, and also regarding why (if peers were not found to be valuable) they were not a good source of PD information. In terms of the valuable contributions of peers to PD, 3 main themes emerged: (i) peers as a source of information (48.9%), (ii) peers offering moral support (18.8%), and (iii) peers initiating PD activities which encouraged the respondents to also participate (6.8%) (Section 7, Q9 in S1 File). Under the first and most prevalent theme, peers were considered a valuable source of information, regarding PD opportunities and events to attend, as well regarding job opportunities and providing help in the job application process. The second most prevalent theme under peer-support was the moral support and sense of camaraderie which peers provided to each other during their time in graduate school. A small percentage of respondents specifically mentioned that their peers were themselves engaged in organizing PD events, and therefore made these opportunities more visible to them and encouraged them to participate. Responses are summarized in Table 3.

**Table 3:** Peer-support in PD (N = 99, 74.4%).

Code	Description of Code	Example Responses
Peers as a source of information (48.9%)	Peers provided valuable information regarding PD opportunities, events, job opportunities, etc.	“My cohort (and especially my group members) were great for sharing **fellowship applications**, up to and including feedback from things like the NRC [National Research Council]. Sharing stories and information about **job interviews** and opportunities provides valuable insight that senior mentors can’t.”
		“It wasn’t until I began talking to other Ph.D. students did I start to notice various **trends in career aspirations** and p**rofessional development goals**.”
Peers offering moral support (18.8%)	Sense of camaraderie is demonstrated through peers being caring and supportive	“Graduate school is taxing in many ways. Your **peers are the ones who understand what you are going through** because they are also going through the same struggles. A spouse or significant other can provide balance and an outlet, but it is more difficult for them to relate to your struggles.“
Peers initiating PD activities which encouraged respondents to participate (6.8%)	Peers organizing events provided more visibility and encouragement for participation	“A lot of **my peers and I are in separate graduate student organizations**. In those organizations we all host professional development workshops and other opportunities open to all graduate students that have really introduced me to career paths and funding opportunities I would not have known about otherwise.“

For those who elaborated on why they did not find their peers of much value in aiding their PD, 19.5% said that their peers were not knowledgeable or experienced enough to be helpful. A quote from one such respondent is provided below.

*“We all knew about the same amount of info*, *so we had some ability to help each other*. *Not enough experience to draw from for advice*.*”*

This is not surprising, as graduate students are in the program to learn and grow professionally, and while they can be a great support to each other, they require access to knowledge sources (especially regarding diverse career paths), skill-development opportunities (workshops as well as immersive experiences such as internships), and interactions with more experienced professionals from whom to learn from (networking opportunities) in order to achieve multi-faceted PD. A small percentage of respondents (6.8%) mentioned that their peers were either not interested in PD or not forthcoming with sharing information (6.0%).

**Fig 6:**
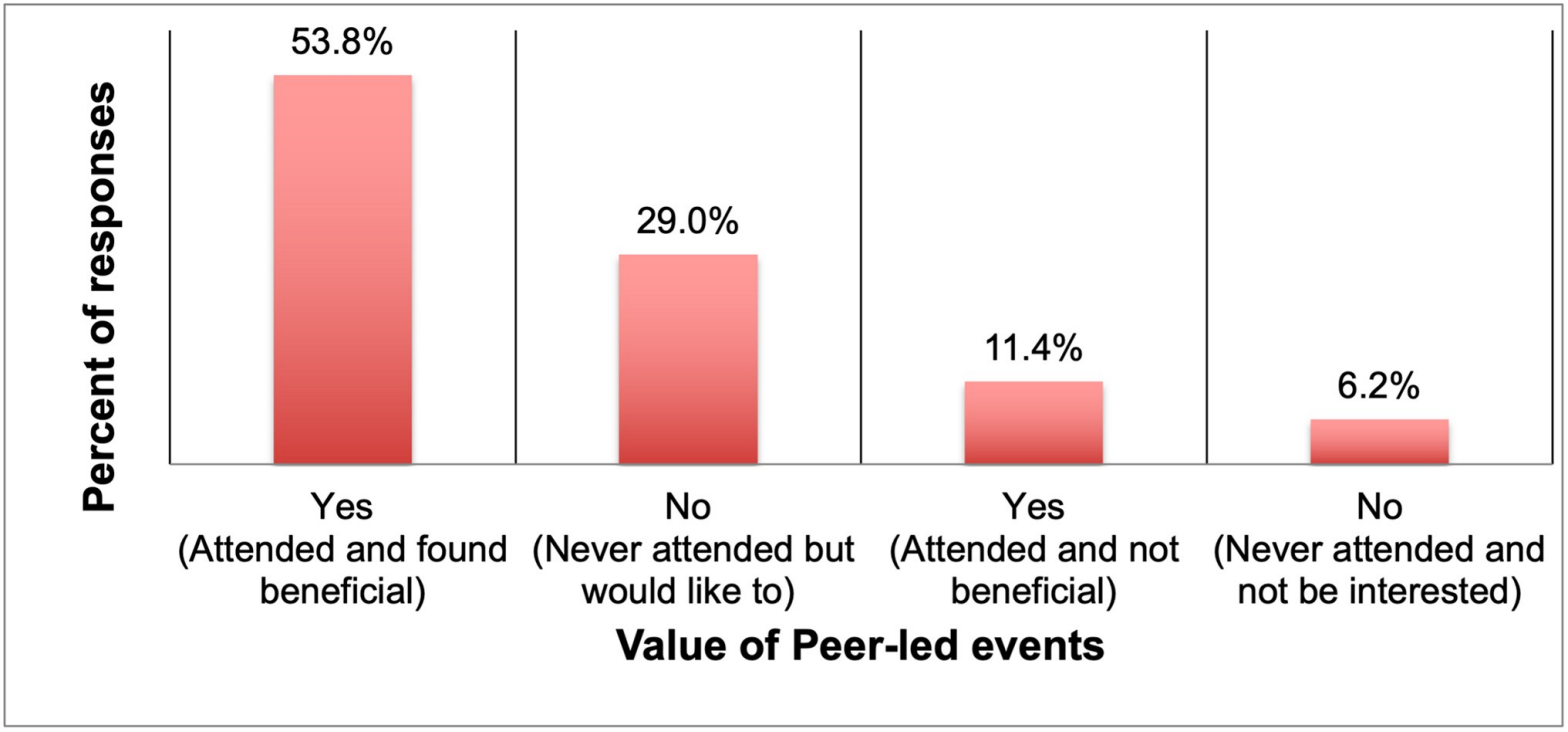
Value of peers-led events in providing information about PD opportunities (n = 173).

When asked if respondents had attended at least one PD event that was organized by their peers that they found to be beneficial; a total of 173 responses were received. Of these, more than half (53.8%) answered “Yes, I have attended such an event and found it beneficial” (Fig 6).

The next highest percentage (29.0%) answered “No, I have never attended such an event but would like to/ have liked to try it out”. Of the 20 people who provided a reason for selecting this option, an overwhelming majority cited that either such peer-led events did not exist at their departments or that they were simply not informed of them. As the majority of respondents had attended peer-led PD events and cited specific ways in which these events benefited them, the authors learnt that peer-led PD events must be quite common and as such an overwhelming majority of respondents had either attended or were open to attending such events.

A small percentage (11.4%) answered “Yes, I have attended such an event but did not find it beneficial”. Of these only 10 respondents shared the reason for their answer choice. These answers mainly fell into 2 themes, most said that the content presented at peer-led events was not relevant to their interests and a small percentage said that they did not learn anything new at such events. The smallest percentage (6.2%) answered “No, I have never attended such an event and would not be interested in it”. Only 5 respondents gave an explanation for selecting this option and their answers fell into one of the following themes: either they felt that they could find the same information from other sources, or they did not expect peer-led events to be relevant to their interests.

Overall, these results show that while peer networks and the knowledge and emotional support from them can be valuable for the PD of Ph.D. students, there is a knowledge and mentorship gap which must be filled externally.

### Section 4: Alumni perspective on doctoral training

The feedback of recent Ph.D. alumni (those who were within 10 years of having graduated with a Ph.D. at the time of answering the questionnaire) on how to improve the PD process for doctoral programs can provide valuable insight to inform the efforts for supporting current Ph.D. students. In this fourth and final section, the authors explored how Ph.D. alumni transitioned from their programs into their respective professional roles and any challenges they faced in this process. Feedback on the most useful PD opportunities during Ph.D. training, as well as suggestions for improvement were also collected, to capture how the Ph.D. training served alumni in their careers post-graduation.

#### Current professional roles of Ph.D. alumni

**Fig 7:**
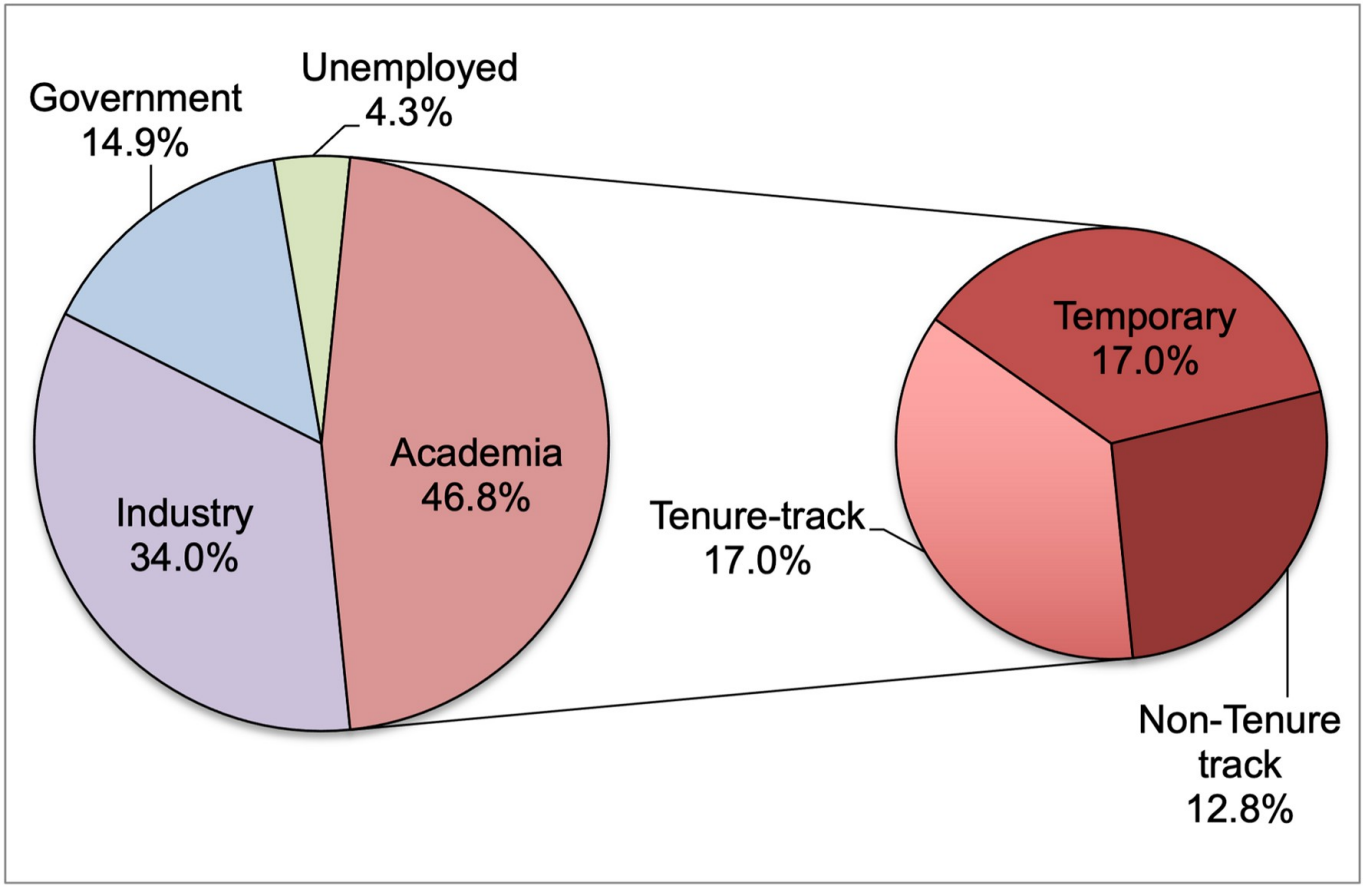
Percentage of alumni respondents (n = 47) within each professional sector, including a breakdown of the type of role within academia.

Within the sample set of alumni respondents, 47 respondents answered the question “Please tell us about your current professional role/designation”. As shown in Fig 7, 46.8% worked within academia and were almost equally distributed among tenure-track, non-tenure-track, and temporary positions within academia. The next highest percentage of respondents, 34.0% were from the industrial sector, followed by 14.9% in the government sector, and 4.3% who reported that they were unemployed at the time of answering the questionnaire. Overall, about half the alumni participating in this study were in academia, but an almost equal proportion were in non-academic positions in other sectors. Even within academia, a sizable proportion (about one third) were in temporary roles; indicating that there would be some shift in their jobs either within academia or a shift to a different career path outside academia. Given this snapshot of post-graduation employment within the respondents and other data about the job market outcomes of STEM Ph.D.s in the U.S. [20, 21], training Ph.D.s exclusively for academic careers is not consistent with the career opportunities and typical outcomes in the job market.

#### Shifts in preferences for career paths post graduation

In order to further probe if the preferences of alumni respondents regarding their career paths had changed since graduating with their Ph.D.s, the respondents were asked if their preferences had changed, and if so, why. Of the 56 alumni respondents who answered this question, 35.7% said yes and 16.1% said that they did not know/ did not have a desired career path in mind while they were in the doctoral program. The remaining 48.2% respondents said that their preferences regarding their career path had remained unchanged after graduation (Section 8, Q2 in S1 File). It is important to note that a little over half the respondents (51.8%) experienced some change or growth in their ideas regarding their career paths after graduation. Within those respondents whose preferences changed (35.7%, 20 respondents), half of them (10 respondents) specifically decided to no longer pursue an academic career path. The major reasons for this change included learning about career paths outside of academia, which they had not previously known about and realizing that an academic career was not worth the effort/ they had had a bad experience in academia. Other reasons for a changed preference for career paths dovetailed into the following themes, some found it too difficult to get into their originally chosen career path, some changed their mind as a result of re-evaluating their personal values such as work-life balance, and some obtained a good job or postdoctoral researcher position by chance, and that changed the course of their career path (Section 8, Q3 in S1 File). Shifts in preferences for career paths away from academia highlight the importance of not only incorporating training within doctoral programs which would be transferable outside academia; but also, the importance of creating awareness about non-academic career paths early on in the Ph.D. training so that students can plan and position themselves to transition into these roles without delays, post-graduation.

The alumni set of respondents were also asked “Are you in your desired career/ job or know the path to getting there?” A total of 34 respondents answered this question, of which 79.4% respondents said yes, 17.6% said maybe, and only 3.0% said no (Section 8, Q4 in S1 File). While a longitudinal study would inform if these respondents continued to feel career satisfaction within their chosen paths and if they choose to make any changes in the future; it is encouraging to note that within 10 years from graduating, most alumni felt that they were in their desired career path or knew how to get there, despite about half of them having changed their preferences regarding career paths after graduation.

#### Transitioning to the first job post-graduation

**Fig 8:**
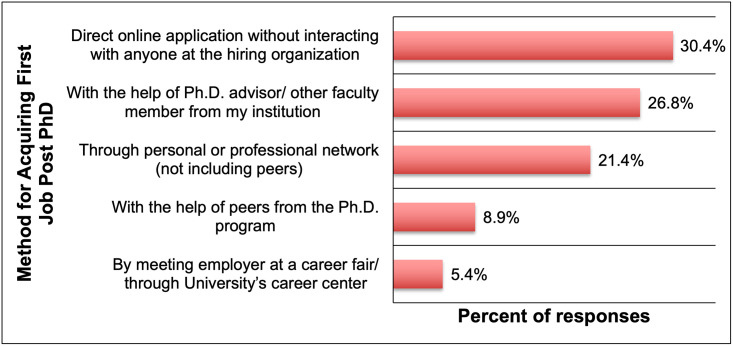
Support for transitioning to the first job after graduation by percentage of alumni responses (n = 56).

When alumni respondents were asked how they got their first position after graduating from the doctoral program (Fig 8), the authors were interested in exploring whether the majority of students tackled the transition independently or if they received support from their doctoral programs. The authors found that just over half of the respondents found their first jobs without direct assistance from their programs; through direct cold applications (30.4%) or their personal or professional networks (21.4%). Of the other half, it was encouraging to note that 26.8% of the respondents obtained their first jobs with the help of their Ph.D. advisor/ other faculty members from their institutions. Some respondents reported having got their first jobs with help from their peers from the Ph.D. program (8.9%) and by meeting their employer at a career fair/ through University’s career center (5.4%).

Upon asking alumni respondents about the most challenging aspects of transitioning from the Ph.D. program to their first job post-graduation, 40 responses were received (Table 4).

**Table 4:** Challenges in transitioning from doctoral program to first position outside the Ph.D. laboratory (N = 40).

Code	Description of Code	Example Responses
Adjusting to unfamiliar environment (45.0%)	Getting used to new organizational structures, team dynamics, expectations from supervisors, and time-management.	“Getting used to the **different environment and expectations**, and finding a better work-life balance than I had as a graduate student.”
		“Adjusting to the **results-based research** approach of doing science.”
		“**Working independently** rather than as part of a group.”
Finding job/ becoming competitive applicant (25.0%)	Challenges associated with not being aware of career options, demonstrating transferability of skills, and lack of professional networks.	“**Proving I had the skills** to be successful in a new context. I am lucky that I was given the **advice to cultivate the necessary skills** while in graduate school because otherwise I could easily have **focused only on my research**, leaving me in no position to pursue my desired career once I completed the program.”
		“Reaching out to **potential employers**.”
		“Getting an in. I sent out ~70 resumes with either **no response or rejection**. It was demoralizing to deal with this.”
Learning new job-related skills (17.5%)	Learning new skills including research and teaching related, as well as business and management related.	“Being a **manager**.”
		“Jumping into a lab with a **new focus and new required skill-set**.”
		“The lack of **business experience**, but this is very specific to my particular case.”
Transitioning to non-academic role (15.0%)	Challenges associated with how non-academic sectors perceive Ph.D.s, lack of awareness among Ph.D.s about the norms of other sectors, and lack of support for non-academic job transitions.	“The most difficult (part) was to **switch from academic to private industry**. Because PhD degrees are badly recognized in my country.”
		“In my case, there was little to no support for applying to jobs that were not faculty jobs at a university. Even the Career Center in the College of Education, where my department was located, only seemed to **focus on jobs in school districts, and mostly for undergrads**. At the time in 2014 (and this may still be the case), there was virtually no help for figuring out other paths and no obvious places to go for help. Even in my department, a lot of the **faculty did not even recognize the existence of non-academic paths—or even career paths as faculty but not at a R1 institution**. It was very frustrating. To this day, I’m not sure whether most of the faculty in my department know or care what I do. Other than my advisor who I am still in some contact with, no one has reached out and I suspect some of them might consider my work not as valuable as theirs.”

The following themes emerged from the responses, with 45.0% indicating that adjusting to an unfamiliar environment was the toughest part. This answer encompassed struggles such as getting used to new organizational structures, team dynamics, expectations from supervisors, and time-management. Some respondents (15.0%) specifically cited challenges associated with transitioning to sectors outside academia, including adapting to research environments in industry which are result-oriented and deadline-driven. While some of these challenges are inevitable for transitioning to and growing in any new role, greater support for internships during the doctoral program could provide students with a broader sense of how research expectations and managerial environments vary between academia and industry, prior to starting their job search. A quarter of the respondents highlighted challenges related to obtaining the first job outside the Ph.D. lab and becoming competitive job applicants. These included not being aware of career options at the time of transition, difficulty in demonstrating transferability of skills acquired through Ph.D. training, and lack of professional networks to help connect with potential employers. These responses further underscored the issue of job-readiness and obtaining a first job which serves not merely as a filler or stop-gap position; but as a meaningful step towards building the career of choice. The last theme which emerged from this set of responses was, learning new job-related skills (mentioned by 17.5% of the respondents) such as business and managerial skills, as well as research and teaching skills (Section 8, Q8 in S1 File).

#### Assessment of PD opportunities in doctoral training

**Fig 9:**
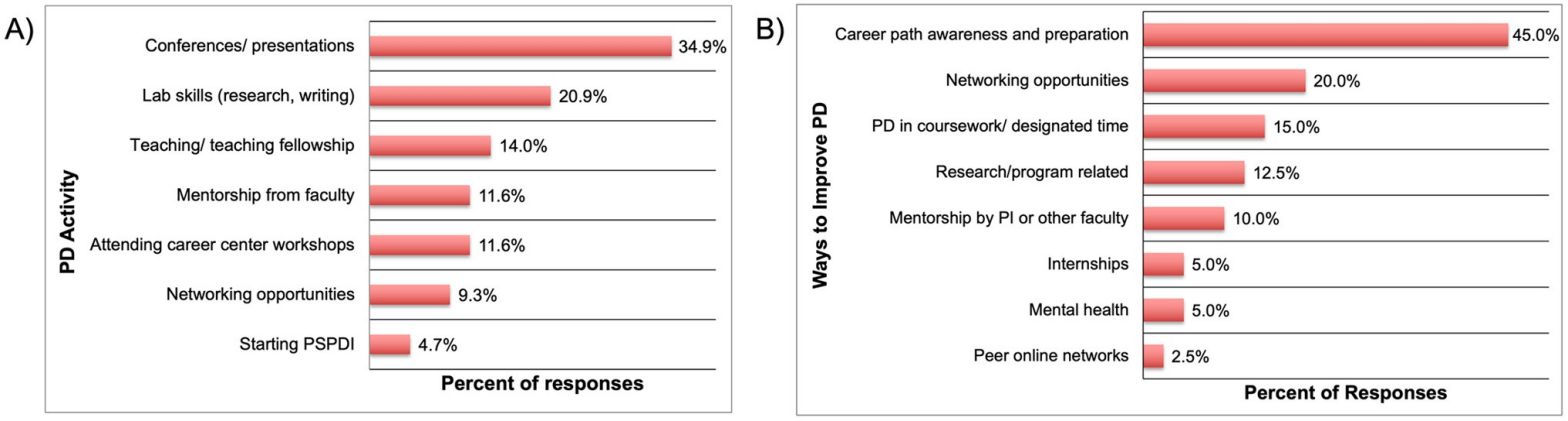
Alumni responses on a) the most valuable PD activities in doctoral training (n = 43), and b) ways to improve PD activities within doctoral training (n = 40).

When alumni respondents were asked what they considered to be the most valuable PD activity from their doctoral experience, 43 responses were received (Fig 9a). Of these, approximately a third of the responses (34.9%) indicated that attending conferences/ making presentations were the most valuable, followed by laboratory skills (such as research and technical writing, 20.9%) and teaching/ teaching fellowships (14.0%). The remaining responses included a range of activities and support systems such as university career centers (11.6%), faculty member support (11.6%), networking (9.3%), and starting a peer-supported professional development initiative (4.7%) (Section 8, Q9 in S1 File).

Interestingly, the top 3 activities mentioned by alumni in response to this question were all directly related to the doctoral program requirements or engagement with broader academic community contexts (tiers 1 and 2, Table 2). It can also be seen that at least two of these activities were generally highly valued by current and former Ph.D. students based on their responses presented in section 2.2; where of the 18 activities presented as options, “Presenting at a national conference” was ranked at number 2 and “Learning a new scientific skill such as an instrumentation technique, computer language, or software” was ranked at number 4. However, “Serving as teaching assistant” was ranked relatively lower at number 9. A number of activities, most of which were farther from the direct context of the Ph.D. program requirements, such as job search (tier 4), expanding networks beyond academia (tier 3), and engaging with broader academic community (tier 2), were ranked above serving as a teaching assistant, when respondents were presented with a broader range of activities to choose from. Therefore, there are a host of PD opportunities valued by Ph.D. students which are currently inaccessible, either due to lack of opportunity or time; and these would be important avenues to explore for improving their PD as part of Ph.D. training.

It is also interesting to note that while “Getting your resume reviewed by a professional in your field/ a career coach” and “Attending an interview skills training workshop” were ranked at number 1 and number 5 respectively in section 2.2, as highly valued PD activities; services offered by university career centers such as workshops and resume support were only cited by 11.6% of the alumni respondents as valuable PD opportunities in their programs. This speaks to the need for taking a closer look at the services offered by university career centers in terms of whether they are accessible to and meeting the needs of the Ph.D. students.

Finally, alumni respondents were asked to suggest ways to improve PD for doctoral students based on their own Ph.D. and job-search experiences. Of the 40 responses received, nearly half of the responses (45.0%) pointed to career path awareness and preparation as the most important area that needed improvement (Fig 9b). Taken together with the fact that more than half of the alumni respondents either did not have any particular path in mind during their doctoral studies or changed their preference about their desired career path after graduation, as well that this change in preference resulted due to increased awareness about career paths outside academia (section 3.3); this points strongly towards the need for career path awareness for Ph.D.s with an emphasis on opportunities beyond academia, such as the private, government, non-profit, and other sectors; including non-R&D roles.

Some respondents indicated the need for building in activities into the doctoral program such as networking (20.0%). It is interesting to note that in Fig 9a as well 9% (n = 44) alumni mentioned networking as the most valuable PD activity while in graduate school. This indicates that networking is clearly valued by alumni respondents, though the exposure to networking opportunities is not always accessible to Ph.D. students.

Another interesting suggestion for improvement was having PD as coursework or having a designated time within the program allocated to PD (15.0%). Respondents elaborated that:

*“Research is demanding on Ph*.*D*. *student’s time*, *which can be a barrier to actually attending different training opportunities”*, *“Time per semester (should be) allotted to mandatory*, *professional development activities”*, *and “It should be something that starts in year 1 and not in the last months of the graduate programme when finishing a thesis is the priority”*.

Some alumni focused on other programmatic aspects of Ph.D. training such as improvements more directly related to developing research skills (12.5%) and opportunities for mentorship by P.I.s or other faculty members (10.0%). The remaining responses touched upon topics such as provisions for mental health support (5.0%), internships (5.0%), and peer online networks (2.5%) (Section 8, Q7 in S1 File).

## Conclusions

The perspectives of current STEM Ph.D. students and recent Ph.D. alumni are important to inform the modernization of Ph.D. programs in order to facilitate job transitions, provide accountability to students, and for program retention. A shift in the employment landscape for Ph.D.s beyond academia has created new challenges for their job transitions post graduation, which are captured in this study through perspectives from recent Ph.D alumni pursuing diverse careers paths. The recent Ph.D. alumni respondents sampled were almost equally distributed in academic and non-academic roles (comprising industry and government jobs).

From the perspective of current STEM Ph.D. students and recent alumni, a key goal of PD during doctoral training is becoming competitive for the job market and ultimately career advancement in a diverse range of paths, including non-academic and non- R&D roles. However, their PD experiences during Ph.D. training are almost exclusively geared towards meeting doctoral program requirements such as developing research and teaching skills and engagement with the broader academic community; than towards expanding their professional networks beyond academia and job search-related experiences (Table 2, Fig 2). While some respondents had access to some tier 3 and tier 4 PD activities (Table 2) through peer-supported initiatives as well as opportunities provided by their program and/ or university; there were additional barriers for students to pursue these experiences. Alumni respondents **identified limited availability of time** due to the demands of the Ph.D. program requirements (such as students prioritizing lab related activities over job-search or PD related activities) **and academic culture** (lack of awareness or encouragement to Ph.D. students for pursuing diverse career paths) as barriers for pursuing tier 3 and 4 activities (Table 2, Fig 2) during doctoral training. In the context of job-search, although their Ph.D. training allowed alumni to develop a robust skillset including transferable skills from research and teaching; some common barriers associated with obtaining a job post-graduation were lack of awareness about career options, limited or no professional networks outside academia, and a lack of preparation and support for non-academic job transitions. Most alumni obtained their first job through cold applications, and some relied on faculty members from their institutions and other personal or professional networks. **Career path awareness is a major gap identified in this study** and is also reflected in the shift or development of career preferences of alumni very late in the program or after graduation, when job realities become apparent. The study also presents perspectives on STEM Ph.D. values and finds that autonomy, challenge, and altruism were identified as the top three career values for Ph.D. students and alumni, as opposed to security, balance, and advancement. It was also noted that the guidance received by Ph.D. students in the form of mentorship and PD came almost exclusively from within the academe—from PIs and peers, and exposure to professionals from outside academia was limited during Ph.D. training. These factors lead to the development of a highly skilled workforce that are often left to discover their career paths on their own and can end-up in stop-gap positions due to a lack of exposure to and preparation for the possible career options available.

### Avenues for improvement

In order to embark on a stable and intentional career path and not get caught in stop gap positions post-graduation; it is critical for Ph.D. students to be aware of the various career options early on in their Ph.D. program and be provided the opportunities and program level support to take active steps in the direction of their desired career path, such as building a professional network, cultivating professional mentors outside academia, and gaining experience through internships; in tandem with building research and teaching skills. The gaps and avenues for improvement identified through this study require a concerted effort from key stakeholders such as, doctoral programs (including P.I.s), research funding agencies, university career centers, professional societies, and the engagement of Ph.D. students themselves; to align the PD in doctoral training with the job-market for Ph.D. graduates. Identifying the specific roles and opportunities for each stakeholder in this process is an important area of investigation, which will be explored by the authors in future studies. Ph.D. students are key drivers of the research enterprise as members who perform studies and experiments which advance knowledge in the STEM fields and make socio-economic contributions to society in a variety of ways post-graduation. Therefore, fostering their success within this ecosystem and creating avenues for their continued socio-economic contributions through viable jobs and careers is necessary in order to maintain and nurture this supply of talent for research.

### Limitations

This study sample includes STEM Ph.D. students and recent Ph.D. graduates from across the globe with the vast majority from the U.S. to whom the results would be most applicable. The STEM fields represented in this study do not reflect all STEM fields proportionately by Ph.D.s produced, and there is higher representation from chemistry, life sciences, and social sciences graduates in this study based on survey uptake. No incentives were provided to those who filled out the questionnaire. As a result, the responses could be from a self-selecting group of individuals who may have a particular interest in PD and / or who may be unsure of pursuing an academic career. This work did not investigate the relationship between barriers to professional development with race, gender, or immigration status. Further, these data were collected before the COVID-19 pandemic and therefore do not reflect changes in the STEM Ph.D. Training and job market. All these considerations may limit the transferability of these results and may not be representative of all STEM Ph.D. students and recent alumni.

## Supporting information

S1 File (DOCX)

## References

[1] Carnevale AP, Smith N, Melton M. STEM and the American Workforce. Georgetown; 2010.

[2] Trapani J, Hale K. Science and Engineering Labor Force. 2019.

[3] Science and Engineering Labor Force. Alexandria, VA; 2018. https://www.nsf.gov/statistics/2018/nsb20181/report/sections/front-matter/about-science-and-engineering-indicators

[4] Gonzalez HB, Kuenzi JJ. Science, Technology, Engineering, and Mathematics (STEM) Education: A Primer. 2012 Aug.

[5] National Center for Science and Engineering Statistics. 2017 Doctorate Recipients from U.S. Universities. In: National Science Foundation [Internet]. 2018 [cited 6 Apr 2019]. https://ncses.nsf.gov/pubs/nsf19301/report/about-this-report

[6] Powell K. The future of the postdoc. Nature. 7 Apr 2015. 10.1038/520144a 25855437

[7] Harsh reality. Nature. 3 Dec 2014. 10.1038/516007b 25471843

[8] McDowell GS, Gunsalus KTW, MacKellar DC, Mazzilli SA, Pai VP, Goodwin PR, et al. Shaping the Future of Research: a perspective from junior scientists. F1000Research. 2015;3. doi: 10.12688/f1000research.5878.2 25653845 PMC4304227

[9] Woolston Chris. THE PRECARITY OF POSTDOCS. Nature. 19 Nov 2020: 505–508.

[10] van Dijk D, Manor O, Carey LB. Publication metrics and success on the academic job market. Curr Biol. 2014;24. doi: 10.1016/j.cub.2014.04.039 24892909

[11] Woolston C. Wheel Of Fortune: Uncertain Prospects for Postdocs. Nature Magazine. 3 Dec 2020: 181–184. 10.1038/d41586-020-03381-3 33262491

[12] Polka JK, Krukenberg KA, McDowell GS. A call for transparency in tracking student and postdoc career outcomes. Mol Biol Cell. 2015;26. doi: 10.1091/mbc.E14-10-1432 25870234 PMC4395122

[13] National Institute of Health. Biomedical Research Workforce Working Group Report. 2012.

[14] St Clair R, Hutto T, MacBeth C, Newstetter W, McCarty NA, Melkers J. The “new normal”: Adapting doctoral trainee career preparation for broad career paths in science. PLoS One. 2017;12: 1–19. doi: 10.1371/journal.pone.0177035 28542304 PMC5443479

[15] Fuhrmann CN, Halme DG, O’Sullivan PS, Lindstaedt B. Improving Graduate Education to Support a Branching Career Pipeline: Recommendations Based on a Survey of Doctoral Students in the Basic Biomedical Sciences. CBE—Life Sci Educ. 2011;10. doi: 10.1187/cbe.11-02-0013 21885820 PMC3164563

[16] Sauermann H, Roach M. Science PhD Career Preferences: Levels, Changes, and Advisor Encouragement. PLoS One. 2012;7. doi: 10.1371/journal.pone.0036307 22567149 PMC3342243

[17] Okahana H. Closing Gaps in Our Knowledge of PhD Career Pathways: Job Changes of PhD Graduates After Earning Their Degree CGS Research in Brief. 2019 Jul.

[18] Zolas N, Goldschlag N, Jarmin R, Stephan P, Smith JO, Rosen RF, et al. Wrapping it up in a person: Examining employment and earnings outcomes for Ph.D. recipients. Science (80-). 2015;350. doi: 10.1126/science.aac5949 26659054 PMC4836945

[19] Turk-Bicakc L, Berger A, Haxton C. The Nonacademic Careers of STEM PhD Holders. 2014 Apr.

[20] Survey of Doctorate Recipients, 2017. National Science Foundation, National Center for Science and Engineering Statistics; http://ncsesdata.nsf.gov/doctoratework/2017/

[21] Langin K. In a first, U.S. private sector employs nearly as many Ph.D.s as schools do. Science. 12 Mar 2019.

[22] Busby BD, Harshman J. Program elements’ impact on chemistry doctoral students’ professional development: a longitudinal study. Chem Educ Res Pract. 2021;22. doi: 10.1039/D0RP00200C

[23] Arnett EM. Formal and informal graduate training for a career in research chemistry. Journal of Chemical Education. 1988. doi: 10.1021/ed065p590

[24] Ph.D. Programs in Chemistry Survey Report. Washington D.C.; 2008. https://www.acs.org/content/dam/acsorg/about/governance/committees/training/reports/cptreports/phd-programs-in-chemistry-survey-report-2008.pdf

[25] Austin AE. Cognitive apprenticeship theory and its implications for doctoral education: a case example from a doctoral program in higher and adult education. Int J Acad Dev. 2009;14. doi: 10.1080/13601440903106494

[26] Glazer EM, Hannafin MJ. The collaborative apprenticeship model: Situated professional development within school settings. Teach Teach Educ. 2006;22. doi: 10.1016/j.tate.2005.09.004

[27] Maher MM, Gilmore JA, Feldon David F., Davis TE. Cognitive Apprenticeship and the Supervision of Science and Engineering Research Assistants. J Res Pract. 2013;9: undefined. Available: http://jrp.icaap.org/index.php/jrp/article/view/354/311

[28] Stephan P. Perverse incentives. Nature. 4 Apr 2012. doi: 10.1038/484029a 22481339

[29] Lorsch JR. Maximizing the return on taxpayers’ investments in fundamental biomedical research. Mol Biol Cell. 2015;26. doi: 10.1091/mbc.E14-06-1163 25926703 PMC4436771

[30] A Data-Based Assessment of Research-Doctorate Programs in the United States (with CD). Washington, D.C.: National Academies Press; 2011. 22379653

[31] Paglis LL, Green SG, Bauer TN. Does adviser mentoring add value? A longitudinal study of mentoring and doctoral student outcomes. Res High Educ. 2006;47. doi: 10.1007/s11162-005-9003-2

[32] Vekkaila J, Virtanen V, Taina J, Pyhältö K. The function of social support in engaging and disengaging experiences among post PhD researchers in STEM disciplines. Stud High Educ. 2018;43: 1439–1453. doi: 10.1080/03075079.2016.1259307

[33] Schillebeeckx M, Maricque B, Lewis C. The missing piece to changing the university culture. Nature Biotechnology. 8 Oct 2013. doi: 10.1038/nbt.2706 24104758

[34] Ritchie TS, Perez Cardenas MT, Ganapati S. Establishment and Implementation of a Peer-Supported Professional-Development Initiative by Doctoral Students, for Doctoral Students. J Chem Educ. 2018;95: 1947–1953. doi: 10.1021/acs.jchemed.8b00337

[35] Gardner SK. “I Heard it through the Grapevine”: Doctoral Student Socialization in Chemistry and History. High Educ. 2007;54. doi: 10.1007/s10734-006-9020-x

[36] Council of Graduate Schools and Educational Testing Service. Pathways Through Graduate School and Into Careers. Report from the Commission on Pathways Through Graduate School and Into Careers. Princeton, NJ; 2012.

[37] Allum JR, Kent JD, McCarthy MT. Understanding PhD Career Pathways for Program Improvement: A CGS Report. Washington D.C.; 2014.

[38] Mitic RR., Okahana H. PhD Professional Development: Value, Timing, and Participation. Washington, D.C.; 2021 Jan.

[39] Nerad M. Professional Development for Doctoral Students: What is it? Why Now? Who does it? Nagoya J High Educ. 2015;15: 285–319.

[40] Sharmini S, Spronken-Smith R. The PhD–is it out of alignment? High Educ Res Dev. 2020;39. doi: 10.1080/07294360.2019.1693514

[41] Ducheny K, Alletzhauser HL, Crandell D, Schneider TR. Graduate student professional development. Prof Psychol Res Pract. 1997;28. doi: 10.1037/0735-7028.28.1.87

[42] Rizzolo S, DeForest AR, DeCino DA, Strear M, Landram S. Graduate Student Perceptions and Experiences of Professional Development Activities. J Career Dev. 2016;43. doi: 10.1177/0894845315587967

[43] Nowell L, Ovie G, Berenson C, Kenny N, Hayden KA. Professional Learning and Development of Postdoctoral Scholars: A Systematic Review of the Literature. Educ Res Int. 2018;2018. doi: 10.1155/2018/5950739 PMC6280376 30518418

[44] Cui Q, Harshman J. Qualitative Investigation to Identify the Knowledge and Skills That U.S.-Trained Doctoral Chemists Require in Typical Chemistry Positions. J Chem Educ. 2020;97: 1247–1255. doi: 10.1021/acs.jchemed.9b01027

[45] Bryan B, Guccione K. Was it worth it? A qualitative exploration into graduate perceptions of doctoral value. High Educ Res Dev. 2018;37. doi: 10.1080/07294360.2018.1479378

[46] Braun V, Clarke V. Using thematic analysis in psychology. Qual Res Psychol. 2006;3. doi: 10.1191/1478088706qp063oa 32100154

[47] Rybarczyk B, Lerea L, Lund PK, Whittington D, Dykstra L. Postdoctoral Training Aligned with the Academic Professoriate. Bioscience. 2011;61. doi: 10.1525/bio.2011.61.9.8

[48] American Chemical Society. The Place of Values in my Job Search. In: Planning My Job Search Learner Application Guide [Internet]. 2009 [cited 6 Mar 2021]. https://acs.learn.com/files/upload/rotator_staging/web/ThePlaceofValuesinmyJobSearch.html

[49] Dawis RV., Lofquist LH. A psychological theory of work adjustment: An individual-differences model and its applications. Minneap Univ Minnesota Press. 1984.

[50] Lent RW, Brown SD, Hackett G. Toward a Unifying Social Cognitive Theory of Career and Academic Interest, Choice, and Performance. J Vocat Behav. 1994;45. doi: 10.1006/jvbe.1994.1027

[51] St. Clair RL, Melkers J, Rojewski J, Ford K, E Dahl T, McCarty NA., et al. Doctoral Trainee Preferences for Career Development Resources: The Influence of Peer and Other Supportive Social Capital. Int J Dr Stud. 2019;14. doi: 10.28945/4436

