# Supplementary material for: Professional development and career-preparedness experiences of STEM Ph.D. students: Gaps and avenues for improvement
Source: PLoS One. 2021 Dec 16;16(12):e0260328. doi: 10.1371/journal.pone.0260328 (PMC8675721; doi:10.1371/journal.pone.0260328)
Supplement: S1 File — (DOCX) [file pone.0260328.s001.docx]

**SUPPORTING INFORMATION**

**Professional development and career-preparedness experiences of STEM Ph.D. students: Gaps and avenues for improvement**

Shweta Ganapati^1‡^ & Tessy S. Ritchie^2‡^*

^1^ Mitacs Canadian Science Policy Fellow 2020-21, Natural Sciences & Engineering Research Council of Canada, Ottawa, Canada

^2^ Department of Chemistry and Life Science, United States Military Academy, West Point, New York, United States of America

*Corresponding author

E-mail: [tessy.ritchie@westpoint.edu](mailto:tessy.ritchie@westpoint.edu)

‡ All authors contributed equally and should be considered co-first authors.

**Survey title:** Professional Development and Career Preparation in Doctoral Programs Survey

**NOTE:** Total valid survey responses were 176. Questions not analyzed were removed.

**Section 1:** Consent Form

ADULT CONSENT TO PARTICIPATE IN A RESEARCH STUDY. Professional Development and Career Preparation in Doctoral Programs.

PURPOSE OF THE STUDY. You are being asked to be in a research study. The purpose of this research is to document experiences relating to professional development and career preparation at the doctoral and postdoctoral level. If you choose to participate, your participation will involve either completing a short survey with the option for a follow up interview.

NUMBER OF STUDY PARTICIPANTS. If you decide to be in this study, you will be one of around 300 people in this research study.

DURATION OF THE STUDY. Your participation will require you to complete an online survey (10-15 minutes) with the option for a follow up interview (45 minutes). PROCEDURES. If you agree to be in the study, we will ask you to do the following things: 1. complete an online survey (10-15 minutes) with the option for a follow up interview (45 minutes).

RISKS AND/OR DISCOMFORTS. There are no known risks associated with this study. BENEFITS. The following benefits may be associated with your participation in this study: Improve the quality of professional development and career preparation at the doctoral and post-doctoral level.

ALTERNATIVES. There are no known alternatives available to you other than not taking part in this study.

CONFIDENTIALITY. The records of this study will be kept private and will be protected to the fullest extent provided by law. In any sort of report, we might publish, we will not include any information that will make it possible to identify a subject. Research records will be stored securely and only the researcher team will have access to the records. However, your records may be reviewed for audit purposes by authorized University or other agents who will be bound by the same provisions of confidentiality.

POTENTIAL FOR USE OF DATA IN FUTURE STUDIES:

Identifiers will be removed from the identifiable private information as part of this research study, and after removal the information could be used for future research studies or distributed to another investigator for future research studies without additional informed consent, as well as used as part of publications.

COMPENSATION & COSTS. You will receive no compensation for your participation. You will not be responsible for any costs to participate in this study.

RIGHT TO DECLINE OR WITHDRAW. Your participation in this study is voluntary. You are free to participate in the study or withdraw your consent at any time during the study. Your withdrawal or lack of participation will not affect any benefits to which you are otherwise entitled. The investigator reserves the right to remove you without your consent at such time that they feel it is in the best interest.

RESEARCHER CONTACT INFORMATION. If you have any questions about the purpose, procedures, or any other issues relating to this research study you may contact Dr. Tessy Ritchie at [ritchie@usna.edu](mailto:ritchie@usna.edu).

IRB CONTACT INFORMATION. If you would like to talk with someone about your rights of being a subject in this research study or about ethical issues with this research study, you may contact the USNA Human Research Protection Program office by phone at 410.293.2513 or by email at HRPPoffice@usna.edu.

**Q1:** Do you consent?

Answer options (N = 176):

- Yes (100%, 176 responses)
- No (only those who consented could proceed to the rest of the survey)

**Section 2:** Doctoral Program Enrollment

**Q1:** Are you currently enrolled in a Ph.D. program?

Answer options (N = 176):

- Yes (67.6%, 119 responses)
- No (32.4%, 57 responses)

**Section 3:** Doctoral Program Enrollment for Current Students

**Q1:** How many years have you been in the doctoral program?

Answer options provides (N = 118)

| **Years in program (currently enrolled)** | **Number of responses** | **Percentage of responses** |
| --- | --- | --- |
| <1 | 16 | 13.6% |
| 1 | 11 | 9.3% |
| 2 | 17 | 14.4% |
| 3 | 20 | 16.9% |
| 4 | 24 | 20.3% |
| 5 | 19 | 16.1% |
| 6 | 7 | 5.9% |
| 7 | 2 | 1.7% |
| 7+ | 2 | 1.7% |

**Section 4:** Doctoral Program Enrollment to filter for Alumni

**Q1:** Were you previously enrolled in a Ph.D. program?

Answer options (N = 57):

- Yes (100%, 57 responses)
- No (0%, 0 responses)

**Section 5:** Previous Doctoral Program Enrollment

**Q1:** How many years did it take you to get your Ph.D.?

Answer options (N= 57):

| **Years in program (alumni)** | **Number of responses** | **Percentage of responses** |
| --- | --- | --- |
| <1 | 0 | 0.0% |
| 1 | 0 | 0.0% |
| 2 | 0 | 0.0% |
| 3 | 2 | 3.5% |
| 4 | 16 | 28.1% |
| 5 | 18 | 31.6% |
| 6 | 12 | 21.1% |
| 7 | 6 | 10.5% |
| 7+ | 3 | 5.3% |

**Section 6:** Doctoral Program Demographic Data

**Q1:** What is/was the general field of study for your Ph.D. work?

Open ended question.

Total number of responses = 176

| **Field of Study** | **Number of responses** | **Percentage of responses** |
| --- | --- | --- |
| Chemistry | 66 | 37.5% |
| Life Sciences | 42 | 23.9% |
| Social Sciences | 37 | 21.0% |
| Psychology | 14 | 8.0% |
| Engineering | 6 | 3.4% |
| Geosciences | 4 | 2.3% |
| Health Sciences | 3 | 1.7% |
| Physics | 3 | 1.7% |
| Math | 1 | 0.6% |

**Q2:** Where is/was your doctoral program’s university located? This is to ensure we have a sample of responses from across the world.

Answer options provided (N = 176)

- In USA (93.2%,164 responses)
- Outside USA (6.8%, 12 responses)

**Q3:** What is your race? Please check all that apply.

Answer options (N = 176)

| **Race** | **Number of responses** | **Percentage of responses** |
| --- | --- | --- |
| White | 120 | 68.2% |
| Asian | 38 | 21.6% |
| Hispanic or Latino | 9 | 5.1% |
| Black or African American | 7 | 4.0% |
| American Indian or Alaska Native | 0 | 0.0% |
| Native Hawaiian or Pacific Islander | 0 | 0.0% |
| Multiracial | 1 | 0.6% |
| Prefer not to answer | 1 | 0.6% |

**Q4:** To which gender do you most identify?

Answer options provided (N = 176)

| **Gender** | **Number of responses** | **Percentage of responses** |
| --- | --- | --- |
| Woman | 110 | 62.5% |
| Man | 66 | 37.5% |
| Other (please type here) | 0 | 0% |
| Prefer not to answer | 0 | 0% |

**Q5:** Please enter the state/US territory your institution is/was located in.

Answer options provided as a standard drop-down menu. (N =161)

| **States** | **Number of responses** | **Percentage of responses** |
| --- | --- | --- |
| Arizona | 1 | 0.6% |
| California | 4 | 2.5% |
| Connecticut | 2 | 1.2% |
| Florida | 3 | 1.9% |
| Georgia | 51 | 31.7% |
| Illinois | 2 | 1.2% |
| Kansas | 1 | 0.6% |
| Massachusetts | 1 | 0.6% |
| Maryland | 68 | 42.2% |
| Michigan | 3 | 1.9% |
| Missouri | 1 | 0.6% |
| Mississippi | 1 | 0.6% |
| North Carolina | 4 | 2.5% |
| New Hampshire | 1 | 0.6% |
| New Mexico | 1 | 0.6% |
| New York | 2 | 1.2% |
| Ohio | 3 | 1.9% |
| Pennsylvania | 1 | 0.6% |
| South Dakota | 1 | 0.6% |
| Tennessee | 1 | 0.6% |
| Texas | 6 | 3.7% |
| Virginia | 1 | 0.6% |
| Wisconsin | 2 | 1.2% |

**Q6:** Please enter the country your institution is/was located in.

Open ended question (N = 176).

| **Countries** | **Number of responses** | **Percentage of responses** |
| --- | --- | --- |
| USA | 164 | 93.2% |
| Canada | 4 | 2.3% |
| Australia | 1 | 0.6% |
| Netherlands | 1 | 0.6% |
| Italy | 1 | 0.6% |
| France | 1 | 0.6% |
| Spain | 1 | 0.6% |
| India | 1 | 0.6% |
| Japan | 1 | 0.6% |
| Slovenia | 1 | 0.6% |

**Section 7:** Professional Development

**Q1:** How would you define professional development?

Open ended question.

Total responses = 176

| **Response** | **Number of responses** | **Percentage of responses** |
| --- | --- | --- |
| Skills   1. Soft skills 2. Technical skills | 91   1. 17 2. 9 | 51.7% |
| Long-term career growth/ planning | 69 | 39.2% |
| Gaining knowledge   1. Career paths | 46   1. 3 | 26.1% |
| Getting job | 34 | 19.3% |
| Experiences | 20 | 11.4% |
| Becoming a competitive job applicant | 14 | 9.7% |
| Networking | 12 | 6.8% |
| Self-improvement | 7 | 4.0% |

**Q2**: Which of the following would you consider as a VALUABLE opportunity for professional development while enrolled in a Ph.D. program? Choose all that apply:

Total responses = 176

| **Activity (value)** | **Number of responses** | **Percentage of responses** |
| --- | --- | --- |
| Getting your resume reviewed by a professional in your field/ a career coach | 151 | 84.4% |
| Presenting at a national conference | 142 | 79.3% |
| Publishing in peer-reviewed journals | 138 | 77.1% |
| Learning a new scientific skill such as an instrumentation technique, computer language, or software | 135 | 75.4% |
| Attending an interview skills training workshop | 134 | 74.9% |
| Attending career seminars where professionals come to discuss their career path | 130 | 72.6% |
| Conducting informational interviews with professionals in your field | 124 | 69.3% |
| Mentoring an undergraduate/junior graduate student in your research lab/ department | 122 | 68.2% |
| Research-funding fellowship seminars where previous fellowship winners give advice on how to draft a successful application including suggestions about personal statements and research proposals | 119 | 66.5% |
| Serving as teaching assistant | 115 | 64.2% |
| Attending receptions at conferences and meetings to form new connections | 112 | 62.6% |
| Doing an internship while in graduate school | 103 | 57.5% |
| Helping a lab-mate advance their research project and/or contributing to their scientific publication | 102 | 57.0% |
| Attending job fairs and company events hosted on your university campus | 94 | 52.5% |
| Attending a thesis writing workshop | 91 | 50.8% |
| Presenting at a small conference hosted at your university with mostly intra-university participation | 89 | 49.7% |
| Serving as a student leader in your department/ at the University | 87 | 48.6% |
| Performing educational outreach efforts at high schools or for young children | 73 | 40.8% |
| Participating in “3-minute thesis” competitions across disciplines | 62 | 34.6% |

**Q3:** Which of the following HAVE YOU DONE while being enrolled in a Ph.D. program? Choose all that apply:

16 options provided; multiple options could be selected.

Total responses = 176

| **Activity (value)** | **Number of responses** | **Percentage of responses** |
| --- | --- | --- |
| Learning a new scientific skill such as an instrumentation technique, computer language, or software | 145 | 81.0% |
| Serving as teaching assistant | 141 | 78.8% |
| Presenting at a national conference | 120 | 67.0% |
| Helping a lab-mate advance their research project and/or contributing to their scientific publication | 120 | 67.0% |
| Publishing in peer-reviewed journals | 118 | 65.9% |
| Mentoring an undergraduate/junior graduate student in your research lab/ department | 114 | 63.7% |
| Attending receptions at conferences and meetings to form new connections | 108 | 60.3% |
| Presenting at a small conference hosted at your university with mostly intra-university participation | 104 | 58.1% |
| Attending career seminars where professionals come to discuss their career path | 92 | 51.4% |
| Serving as a student leader in your department/ at the University | 71 | 39.7% |
| Getting your resume reviewed by a professional in your field/ a career coach | 67 | 37.4% |
| Performing educational outreach efforts at high schools or for young children | 61 | 34.1% |
| Attending a thesis writing workshop | 52 | 29.1% |
| Conducting informational interviews with professionals in your field | 46 | 25.7% |
| Research-funding fellowship seminars where previous fellowship winners give advice on how to draft a successful application including suggestions about personal statements and research proposals | 45 | 25.1% |
| Attending job fairs and company events hosted on your university campus | 41 | 22.9% |
| Attending an interview skills training workshop | 31 | 17.3% |
| Doing an internship while in graduate school | 20 | 11.2% |
| Participating in “3-minute thesis” competitions across disciplines | 17 | 9.5% |

**Q4:** Which of the following do you value in your career – pick top 3

Total responses = 176

| **Value**  **(Provided as options)** | **Number of times selected** | **Category** | **Total for Category** | **Percentage for Category** |
| --- | --- | --- | --- | --- |
| Independence to direct projects and/or make decisions regarding projects | 76 | Autonomy (the desire for freedom and ability to be self-directed) | 109 | 61.9% |
| Supervising and/or leading a team | 33 |  |  |  |
| Performing intellectually demanding tasks | 79 | Challenge (the drive to overcome obstacles and solve difficult problems) | 105 | 59.7% |
| Performing new and different tasks constantly | 26 |  |  |  |
| Doing work that makes a positive difference in society | 81 | Altruism (the opportunity to contribute to the welfare of others) | 100 | 56.8% |
| Helping less privileged members of society | 19 |  |  |  |
| Flexibility in work schedule | 76 | Balance (the desire for equilibrium between personal and business) | 80 | 45.5% |
| Being able to work remotely | 4 |  |  |  |
| Monetary compensation | 54 | Advancement (the need for an opportunity for promotion and recognition) | 73 | 41.5% |
| Receiving recognition | 19 |  |  |  |
| Job security | 55 | Security (the need for stability and predictability) | 61 | 34.7% |
| Having predictability in assigned tasks | 6 |  |  |  |

**Q5:** Of the following options, whom do you MOST prefer talking to for career-related advice?

Total responses = 174

| **Option provided** | **Number of times selected** | **Percentage of selections** |
| --- | --- | --- |
| Entry-level professional | 57 | 32.8% |
| Entry-level alumni | 19 | 10.9% |
| Senior professional | 75 | 43.1% |
| Senior professional alumni | 23 | 13.2% |

**Q6:** Please explain the reason for your selection in the previous question "Of the following options, whom do you MOST prefer talking to for career-related advice?"

Open ended question.

Total responses = 162

**Entry-level alumni = 18**

| Easy to relate to (e.g., closer to age = 3) | 16 |
| --- | --- |
| Current information (Most relevant/ up to date) | 9 |
| Won't judge me | 3 |
| Already know them/ related to them | 3 |
| Most likely to help because they relate to me | 2 |
| Early career = Advice, Senior = Job | 1 |

**Entry-level professional = 55**

| Current information (Most relevant/ up to date) | 37 |
| --- | --- |
| Easy to relate to (e.g., closer to age = 4) | 31 |
| Won't judge me | 3 |
| N/A | 3 |
| Most likely to help | 1 |
| Early career = Advice, Senior = Job | 1 |
| Reliable | 1 |

**Senior Professional alumni = 21**

| Experience | 15 |
| --- | --- |
| Relatable | 9 |
| Breadth of knowledge | 6 |
| Connections/Network | 3 |
| Already know them/ related to them | 3 |
| Likely to be able to hire someone | 1 |
| Reliable | 1 |

**Senior professional = 68**

| Experience | 52 |
| --- | --- |
| Breadth of knowledge | 32 |
| Likely to be able to hire someone | 6 |
| Knows measures of success | 5 |
| Early career = Advice, Senior = Job | 3 |
| Influential = Connections/Network | 3 |
| Relatability/ connection to alumni/people they already know | 3 |
| More reliable | 2 |
| N/A | 2 |

**Q7:** Do you have a professional mentor? If yes, please tell us in general terms how they are related to you/how you met them (e.g.: PI, senior colleague, peer, etc.)

Open ended question.

Total responses = 154

| **Response** | **Number of responses** | **Percentage of responses** |
| --- | --- | --- |
| PI Only | 53 | 34.4% |
| No Mentor | 36 | 23.4% |
| Other Professionals (outside school) | 27 | 17.5% |
| PI + Others | 17 | 11.0% |
| Professors (includes academic advisers/ grad advisers/ major adviser) | 15 | 9.7% |
| Peers in Grad School | 5 | 3.2% |
| Did not specify who | 1 | 0.6% |

**Q8:** How VALUABLE are/were your PEERS (from the Ph.D. program) when it involves/involved learning about new professional development opportunities?

Total responses = 175

| **Options provided** | **Number of times selected** | **Percentage of responses** |
| --- | --- | --- |
| Extremely Valuable | 38 | 21.6% |
| Very Valuable | 51 | 29.0% |
| Somewhat Valuable | 56 | 32.0% |
| Slightly Valuable | 20 | 11.4% |
| Not Valuable | 10 | 5.7% |

**Q9:** Please explain your answer choice from the previous question.

Open ended question.

Total responses = 133

| **Response** | **Number of responses** | **Percentage of responses** |
| --- | --- | --- |
| Source of Information | 65 | 48.9% |
| Supportive/Caring | 25 | 18.8% |
| Peers were involved in PD activities | 9 | 6.8% |
| Passive = not interested in PD | 9 | 6.8% |
| Did not know enough to be helpful | 26 | 19.5% |
| Did not share information | 8 | 6.0% |
| N/A | 3 | 2.3% |
| Limited Interaction | 3 | 2.3% |
| Other Sources were more useful | 1 | 0.8% |
| Valuable, but nonspecific | 2 | 1.5% |
| Not valuable, but nonspecific | 2 | 1.5% |
| Varies among peers, so not that valuable | 2 | 1.5% |
| moderate, but nonspecific | 1 | 0.8% |

**Q10:** Have you attended at least one professional development event that was ORGANIZED by your PEERS (from the Ph.D. program) that you found to be beneficial? For example, career seminars hosting professionals, networking events, etc.

Total responses = 173

| **Options provided** | **Number of times selected** | **Percentage of responses** |
| --- | --- | --- |
| Yes, I have attended such an event and found it beneficial | 93 | 53.8% |
| Yes, I have attended such an event but did not find it beneficial | 18 | 11.4% |
| No, I have never attended such an event but would like to/ have liked to try it out | 51 | 29.0% |
| No, I have never attended such an event and would not be interested in it | 11 | 6.2% |

**Q11:** Please explain your answer choice from the previous question.

Open ended question.

Total responses = 107

| **Yes attended & Beneficial (Total Valid Responses)** | **46** |
| --- | --- |
| Got involved in PD | 9 |
| Allowed networking | 8 |
| Learned about different roles | 10 |
| Skills/Interview Process | 14 |
| Research/Graduate Student Success events | 12 |

| **Yes attended & Not Beneficial (Total Valid Responses)** | **10** |
| --- | --- |
| Did not learn much | 2 |
| Content was not relevant to my interests | 7 |
| Peer group was unsupportive | 1 |

| **Did not attend But Would Like to Try** | **20** |
| --- | --- |
| Events did not exist/ did not know about events | 17 |
| Peer events may not be as beneficial (need non-peers) | 2 |
| Not convenient | 1 |

| **No & Not Interested** | **5** |
| --- | --- |
| Can learn from other sources | 2 |
| Not interesting/ relevant | 2 |
| Not necessary | 1 |

**Section 8:** Alumni Questions

**Note:** The next few questions are for recent Ph.D. graduates (2009 and later).

**Q1**: Did you graduate with your Ph.D. within the last ten years? (2009 or later graduation date).

Options provided: Yes/ No

Total responses = 56

**Q2:** Think back to when you were in graduate school, did your opinions on a desired career path change since graduating?

Total responses = 56

| **Options provided** | **Number of responses** | **Percentages of responses** |
| --- | --- | --- |
| Yes | 20 | 35.7% |
| No | 27 | 48.2% |
| I did not know/did not have a desired career path in mind while I was in the doctoral program | 9 | 16.1% |

**Q3:** Why do you think your opinions on a desired career path changed since graduating?

Open ended question.

Total responses = 20

| **Responses** | **Number of responses** | **Percentage of responses** |
| --- | --- | --- |
| Learned about career paths outside of academia | 6 | 30.0% |
| Discovered that an academic career is not worth the effort/ had a bad experience in academia | 4 | 20.0% |
| Too difficult to get into that career path | 3 | 15.0% |
| Re-evaluation of personal values- work life balance etc. | 3 | 15.0% |
| Went into a good postdoc/job (by chance) | 3 | 15.0% |
| Lost interest in research | 1 | 5.0% |
| N/A | 2 | 10.0% |

**Q4:** Are you in your desired career/ job or know the path to getting there?

Open ended question.

Total responses = 34

| **Options provided** | **Number of responses** | **Percentage of responses** |
| --- | --- | --- |
| Yes | 27 | 79.4% |
| Maybe | 6 | 17.6% |
| No | 1 | 3.0% |

**Q5:** Please tell us about your current professional role/designation.

Open ended question.

Total responses = 47

| **Responses** | **Number of responses** | **Percentage of responses** |
| --- | --- | --- |
| Academia - Tenure Track | 8 | 17.0% |
| Academia - Temporary | 8 | 17.0% |
| Academia - Non-Tenure Track | 6 | 12.8% |
| Industry | 16 | 34.0% |
| Government | 7 | 14.9% |
| Unemployed | 2 | 4.3% |

**Q6:** How did you get your FIRST position after the doctoral program?

Total responses = 56

| **Option provided** | **Number of responses** | **Percentage of responses** |
| --- | --- | --- |
| Direct online application without interacting with anyone at the hiring organization | 17 | 30.4% |
| With the help of Ph.D. advisor/ other faculty member from my institution | 15 | 26.8% |
| Through personal or professional network (not including peers) | 12 | 21.4% |
| With the help of peers from the Ph.D. program | 5 | 8.9% |
| By meeting employer at a career fair/ through University’s career center | 3 | 5.4% |
| I have graduated and am still looking for my first position | 1 | 1.8% |
| Already had a job during PhD | 2 | 3.6% |
| Other | 1 | 1.8% |

**Q7:** Based on your Ph.D. journey, can you think of any way to improve the professional development training available for doctoral students?

Open ended question.

Total responses = 40

| **Responses** | **Number of responses** | **Percentage of responses** |
| --- | --- | --- |
| Career Path Awareness and Preparation | 18 | 45.0% |
| Networking Opportunities | 8 | 20.0% |
| PD in coursework/ designated time | 6 | 15.0% |
| Research/Program Related | 5 | 12.5% |
| Mentorship by PI or Other Faculty | 4 | 10.0% |
| Mental Health | 2 | 5.0% |
| Internships | 2 | 5.0% |
| Peer Online Networks | 1 | 2.5% |

**Q8:** In your experience, what was the most challenging aspect of transitioning from the doctoral program to your FIRST position outside of your Ph.D. laboratory?

Open ended question.

NEW: Total responses = 40

| **Responses** | **Number of responses** | **Percentage of responses** |
| --- | --- | --- |
| Adjusting to unfamiliar environment | 18 | 45.0% |
| Finding job/ becoming competitive applicant | 10 | 25.0% |
| Learning new job-related skills | 7 | 17.5% |
| Transitioning to non-academic role | 6 | 15.0% |

**Q9:** What was the MOST VALUABLE professional development opportunity in your doctoral experience which you benefited from?

Open ended question.

Total responses = 43

| **Responses** | **Number of responses** | **Percentage of responses** |
| --- | --- | --- |
| Conferences/ Presentations | 15 | 34.9% |
| Lab Skills (research, writing) | 9 | 20.9% |
| Teaching/ Teaching Fellowship | 6 | 14.0% |
| Career Center (Resume, workshops) | 5 | 11.6% |
| Faculty | 5 | 11.6% |
| Networking | 4 | 9.3% |
| Starting PSPDI | 2 | 4.7% |
| Other | 3 | 7.0% |
